# Identification of IGFBP2 and IGFBP3 As Compensatory Biomarkers for CA19-9 in Early-Stage Pancreatic Cancer Using a Combination of Antibody-Based and LC-MS/MS-Based Proteomics

**DOI:** 10.1371/journal.pone.0161009

**Published:** 2016-08-31

**Authors:** Toshihiro Yoneyama, Sumio Ohtsuki, Kazufumi Honda, Makoto Kobayashi, Motoki Iwasaki, Yasuo Uchida, Takuji Okusaka, Shoji Nakamori, Masashi Shimahara, Takaaki Ueno, Akihiko Tsuchida, Naohiro Sata, Tatsuya Ioka, Yohichi Yasunami, Tomoo Kosuge, Takashi Kaneda, Takao Kato, Kazuhiro Yagihara, Shigeyuki Fujita, Wilber Huang, Tesshi Yamada, Masanori Tachikawa, Tetsuya Terasaki

**Affiliations:** 1 Division of Membrane Transport and Drug Targeting, Graduate School of Pharmaceutical Sciences, Tohoku University, Sendai, Japan; 2 Department of Pharmaceutical Microbiology, Faculty of Life Sciences, Kumamoto University, Kumamoto, Japan; 3 Division of Chemotherapy and Clinical Research, National Cancer Center Research Institute, Tokyo, Japan; 4 Japan Agency for Medical Research and Development (AMED) CREST, Tokyo, Japan; 5 Division of Epidemiology, Research Center for Cancer Prevention and Screening, National Cancer Center, Tokyo, Japan; 6 Department of Hepatobiliary and Pancreatic Oncology, National Cancer Center Hospital, Tokyo, Japan; 7 Departments of Hepato-Biliary-Pancreatic Surgery, Osaka National Hospital, National Hospital Organization, Osaka, Japan; 8 Department of Oral Surgery, Osaka Medical College, Osaka, Japan; 9 Department of Gastrointestinal and Pediatric Surgery, Tokyo Medical University, Tokyo, Japan; 10 Department of Surgery, Jichi Medical University, Tochigi, Japan; 11 Department of Hepatobiliary and Pancreatic Oncology, Osaka Medical Center for Cancer and Cardiovascular Diseases, Osaka, Japan; 12 Islet Institute, Fukuoka University, Fukuoka, Japan; 13 Hepatobiliary and Pancreatic Surgery Division, National Cancer Center Hospital, Tokyo, Japan; 14 Department of Radiology, Nihon University School of Dentistry at Matsudo, Chiba, Japan; 15 Department of Oral Implant, Nihon University School of Dentistry at Matsudo, Chiba, Japan; 16 Department of Oral Surgery, Saitama Cancer Center, Saitama, Japan; 17 Department of Oral and Maxillofacial Surgery, Wakayama Medical University, Wakayama, Japan; 18 Abnova, Taipei City, Taiwan; Pacific Northwest National Laboratory, UNITED STATES

## Abstract

Pancreatic cancer is one of the most lethal tumors, and reliable detection of early-stage pancreatic cancer and risk diseases for pancreatic cancer is essential to improve the prognosis. As 260 genes were previously reported to be upregulated in invasive ductal adenocarcinoma of pancreas (IDACP) cells, quantification of the corresponding proteins in plasma might be useful for IDACP diagnosis. Therefore, the purpose of the present study was to identify plasma biomarkers for early detection of IDACP by using two proteomics strategies: antibody-based proteomics and liquid chromatography-tandem mass spectrometry (LC-MS/MS)-based proteomics. Among the 260 genes, we focused on 130 encoded proteins with known function for which antibodies were available. Twenty-three proteins showed values of the area under the curve (AUC) of more than 0.8 in receiver operating characteristic (ROC) analysis of reverse-phase protein array (RPPA) data of IDACP patients compared with healthy controls, and these proteins were selected as biomarker candidates. We then used our high-throughput selected reaction monitoring or multiple reaction monitoring (SRM/MRM) methodology, together with an automated sample preparation system, micro LC and auto analysis system, to quantify these candidate proteins in plasma from healthy controls and IDACP patients on a large scale. The results revealed that insulin-like growth factor-binding protein (IGFBP)2 and IGFBP3 have the ability to discriminate IDACP patients at an early stage from healthy controls, and IGFBP2 appeared to be increased in risk diseases of pancreatic malignancy, such as intraductal papillary mucinous neoplasms (IPMNs). Furthermore, diagnosis of IDACP using the combination of carbohydrate antigen 19–9 (CA19-9), IGFBP2 and IGFBP3 is significantly more effective than CA19-9 alone. This suggests that IGFBP2 and IGFBP3 may serve as compensatory biomarkers for CA19-9. Early diagnosis with this marker combination may improve the prognosis of IDACP patients.

## Introduction

Pancreatic cancer is one of the most lethal tumors, with a five-year survival rate of 6% [1]. Currently available biomarkers for pancreatic cancer, such as carbohydrate antigen 19–9 (CA19-9), do not have a sufficient ability to detect pancreatic cancer at an early stage [2]. Therefore, to improve the prognosis of pancreatic cancer, new markers able to identify early-stage pancreatic cancer and (or) the risk diseases for pancreatic cancer are urgently needed [3]. Many mass spectrometry (MS)-based proteomic (discovery-based quantitative proteomics) studies in plasma or serum have been conducted to find such biomarkers [4–6]. However, the wide dynamic range of plasma protein expression and interference by abundant plasma proteins are critical issues for biomarker discovery [7]. In order to detect less abundant candidates, recent MS-based biomarker studies have tended to focus on extending the comprehensiveness of analysis by using various sample concentration methods, such as isoelectric focusing electrophoresis and immunoaffinity depletion of highly abundant proteins [8]. This is because the dynamic concentration range of plasma proteins is over 10 orders of magnitude [9], and an enormous number of proteins, more than one million including isoforms and post-translation modifications, exists in humans [10]. However, it remains difficult to identify effective markers among such an enormous number of candidates from limited numbers of clinical samples due to the low throughput of proteomic analysis. In fact, only a few biomarker candidate protein identified by proteomics have been investigated for potential clinical utility [11]. For these reasons, an alternative strategy is needed to discover new biomarkers for pancreatic cancer.

The potential pool of biomarkers originates from pancreatic cancer cells, and thus compounds leaked or released from pancreatic cancer could be good markers for diagnosis. For instance, CA19-9 was reported to be released from pancreatic cancer tissue [12]. Nakamura *et al*. identified 260 genes that were upregulated in invasive ductal adenocarcinoma of pancreas (IDACP) cells compared to normal pancreatic ductal epithelial cells by using a genome-wide cDNA microarray technique combined with laser microbeam microdissection [13]. Therefore, these genes are potential biomarker candidates for IDACP, and quantification of the proteins derived from these genes in plasma might be useful for IDACP diagnosis. However, screening these biomarker candidates in plasma would require a highly sensitive, high-throughput quantification method able to screen multiple markers in large numbers of samples, since proteins leaked or released from cancer cells are likely to be present at low concentrations [14]. Reverse-phase protein array (RPPA) technology enables highly sensitive protein detection with antibodies [15]. Furthermore, its multiplex capability makes it possible to identify marker candidates using data from several hundred plasma samples. Therefore, RPPA was expected to be applicable for this purpose [15]. In the RPPA format, plasma samples are spotted on a slide tagged with antibodies for target proteins, and the target proteins can be measured and directly compared across multiple samples with low sample volumes [16]. Our quantification system using RPPA is able to quantify a maximum of 384 samples simultaneously, and has already been used to identify plasma biomarkers for detecting cancer and predicting appropriate cancer treatment [16–19].

But, despite its advantages in sensitivity and multiplex ability, RPPA suffers from low specificity in detection and high variation in quantification. Hence, accurate and reliable absolute quantification is necessary to validate biomarker candidates identified by RPPA screening. Antibody-based absolute quantification methods, such as enzyme-linked immunosorbent assay (ELISA), in which two specific antibodies for target protein are usually used, are widely employed for protein quantification, but they have significant disadvantages. For example, as ELISA is a monoplex assay, large amounts of serum or plasma may be needed for multi-marker analysis. Moreover, the quantitative values may vary depending on protein structure (e.g., free, complex and fragmented forms), because the affinity of the antibody for each form may be different. On the other hand, protein quantification by LC-MS/MS with selected reaction monitoring or multiple reaction monitoring (SRM/MRM) is a powerful tool for absolute protein quantification of multiple proteins in very small amounts of serum or plasma. Moreover, LC-MS/MS-based quantification is not dependent on protein structure, because the proteins are completely digested with enzymes. The sensitivity of LC-MS/MS with SRM/MRM is lower than that of RPPA [15], but the specificity is higher, because of the two-stage mass fragment selection in SRM/MRM. Because of these advantages, LC-MS/MS with SRM/MRM is an attractive quantification methodology for biomarker proteins in plasma, and several studies have reported quantification of lower-abundance proteins in non-depleted plasma or serum samples [20–24]. We have developed *in silico* selection criteria for selecting appropriate tryptic peptides to quantify target proteins using SRM/MRM analysis [25,26]. These criteria enable us to select a suitable target peptide within 10 min from protein sequence information alone [27]. On the other hand, there are some time-consuming aspects of LC-MS/MS analysis, such as sample preparation, LC-MS/MS measurement and analysis of the results, which may be problematic for large-scale quantification of biomarker candidates. Here, we overcame these problems by developing a high-throughput SRM/MRM method, employing automated sample preparation, micro LC and an auto analysis system.

Thus, the purpose of the present study was to identify biomarkers for early detection of IDACP by using a new strategy, i.e., the combination of antibody-based proteomics and LC-MS/MS-based proteomics using our newly developed high-throughput SRM/MRM method. With this approach, we were able to quantify biomarker candidates in nearly 600 plasma samples from patients with stage I and II IDACP patients, as well as other diseases, and healthy controls. Our results indicate that the combination of CA19-9, IGFBP2 and IGFBP3 is more effective than CA19-9 alone for diagnosis of IDACP.

## Materials and Methods

### Materials

The stable isotope-labeled peptides and unlabeled peptides listed in S1 Table were synthesized at Thermoelectron Corporation (Sedantrabe, Germany). The concentrations of peptide solutions were determined by quantitative amino acid analysis, using an HPLC-UV system with post-column ninhydrin derivatization (LaChrom Elite, Hitach, Tokyo, Japan). Standard human serum was purchased from Sigma Chemical Co. (St. Louis, MO). All other reagents were commercial products of analytical grade.

### Ethics statement

This study was done with written informed consent from every subject. The research protocols for the present study were reviewed and approved by the ethics committees of the National Cancer Research Institute (Number 20–003 and 21–140), Faculty of Life Sciences, Kumamoto University (Number 945) and the Graduate School of Pharmaceutical Sciences, Tohoku University (Number 12–12).

### Plasma samples

Plasma samples for RPPAs (set for RPPAs) and LC-MS/MS analysis (early-stage set and all-stage set) listed in Table 1 were collected from seven medical institutions as part of the “Third-Term Comprehensive Control Research for Cancer” project and stored at -80°C at the National Cancer Center Research Institute until analysis as described previously [17,18,28]. All study subjects were finally diagnosed and classified at each institution before RPPA and LC-MS/MS analysis were performed. Plasma samples were collected across all seven institutions between 2006 and 2008 according to Standard Operating Procedures (SOP) reported previously [28]. The clinical stages were classified according to the TNM classification of the Union for International Cancer Control. Patients with a history of cancer or with other diseases were excluded. Healthy controls were people who had visited a clinic for a benign disease or other treatment, such as an implant or gum disease. The selection of sample size to give sufficient statistical power for biomarker validation was based on previous reports [14,29]. Plasma samples were taken before treatment. For the RPPA set, researchers were not blinded to the clinical characteristics of the samples and samples were not age-matched, because RPPA was performed only for the purpose of biomarker screening. In the validation of the biomarker candidates, researchers were blinded to the clinical characteristics of the early-stage set and all-stage set prior to LC-MS/MS analysis and samples were age-matched. Sample sets were determined by K.H. and M.K., and LC-MS/MS analyses were performed by T.Y. and S.O., who have previously conducted more than 100 LC-MS/MS analyses of plasma samples and identified hydroxylatedα-fibrinogen as a biomarker for pancreatic cancer [30]. Forty-two plasma samples for comparing the quantitative values of C2 and IGFBP2 between RPPA and LC-MS/MS were also collected as part of the “Third-Term Comprehensive Control Research for Cancer” project. Subjects who provided 125 plasma samples used for comparing the quantitative values of CRP, IGFBP3 and adiponectin between antibody-based absolute quantification methods (immunoturbidimetry and ELISA) and LC-MS/MS were among the participants in validation studies of a semi-quantitative food frequency questionnaire (FFQ) used for a case-control study of colorectal adenoma in Tokyo [31,32, 33–35]. CA19-9, carcinoembryonic antigen (CEA) and Duke pancreatic monoclonal antibody type 2 (DUPAN-2) were measured at SRL Co. (Tokyo, Japan). The quantitative values of CRP, IGFBP3 and adiponectin determined by immunoturbidimetry or ELISA were the same data reported previously [35].

**Table 1:** Characteristics of subjects.

Subject Name			N =			Age (Years), Mean ± SD
			Total	Male	Female	
Set for RPPAs						
	Healthy controls		106	71	35	42.4 ± 15.3
	IDACP		164	98	66	65.3 ± 9.5
	Chronic pancreas		10	8	2	57.7 ± 13.0
	Hepatocellular carcinoma		11	9	2	70.3 ± 5.8
	Cholangiocarcinoma		13^a^	9	3	67.3 ± 9.5
	Gastric cancer		30	25	5	64.0 ± 10.3
	Colon cancer		28	14	14	63.3 ± 12.0
Early-stage set						
	Healthy controls		65	30	35	66.2 ± 5.6
	IDACP		38	24	14	68.6 ± 7.5
All-stage set						
	Healthy controls		38	22	16	60.2 ± 12.3
	IDACP		101	60	41	65.5 ± 9.6
	Pancreatic diseases					
		MCNs	5	0	5	56.2 ± 21.7
		IPMNs	25	15	10	68.1 ± 7.5
		Endocrine neoplasms	11	3	8	61.3 ± 5.8
		Chronic pancreatitis	3	2	1	61.3 ± 2.1
		Others	6	4	2	61.3 ± 15.1
	Esophagus cancer		10	9	1	64.1 ± 9.7
	Gastric cancer		119	84	35	66.4 ± 10.9
	Cholangiocarcinoma		24	14	10	69.4 ± 10.7
	Hepatocellular carcinoma		12	10	2	70.6 ± 5.6
	Colon cancer		127	76	51	64.3 ± 11.0
	Duodenal cancer		10	5	5	69.8 ± 7.9

IDACP, invasive ductal adenocarcinoma of the pancreas; SCNs, serous cystic neoplasms; MCNs, mucinous cystic neoplasms; IPMNs, intraductal papillary-mucinous neoplasms. The number of subjects for RPPAs in each clinical stage of IDACP, classified according to Union for International Cancer Control, is as follows: I (n = 6), II (n = 31), III (n = 44), IV (n = 88). The number of patients in each clinical stage of IDACP for early-stage set is as follows; I (n = 4), II (n = 34). The number of patients in each clinical stage of IDACP for all stage set is as follows; I (n = 4), II (n = 19), III (n = 26), IV (n = 51) and unknown (n = 1).

^a^ Sex and age of one patient were not recorded.

### RPPAs

RPPAs using 130 antibodies, shown in S2 Table, were performed as described previously [17,19]. Plasma samples were serially diluted (8-, 16-, 32- and 64-fold) and printed onto ProteoChip glass slides (Proteogen, Seoul, Korea) in quadruplicate using a protein microarrayer robot (Kakengeneqs, Matsudo, Japan) at 4°C. The spotted slides were blocked with PBS containing 0.5% casein for 60 min and incubated overnight with the antibodies listed in S2 Table. After washing, the slides were incubated with the appropriate biotinylated second antibodies (BA-2000, Vector Laboratories, Burlingame, CA, USA) and subsequently with streptavidin-horseradish peroxide conjugate (GE Healthcare). The peroxidase activity was detected using the Tyramide Signal Amplification (TSA) Cyanine 5 System (Perkin Elmer, Boston, MA, USA) according to the manufacturer’s instructions.

### Automated sample preparation for LC-MS/MS analysis

Five-fold-diluted plasma samples and standard human serum were applied to 96-well microplates (max; 2 plates, 192 samples), and transferred to the Hamilton Microlab STARplus (Hamilton Robotics, Reno, NV). Ten-microliter aliquots of 5-fold-diluted samples, corresponding to 2 μL plasma, were solubilized in 15 μL of 8 M urea in 100 mM Tris-HCl (pH 8.5), and S-carbamoylmethylated with 5 μL of 20 mg/mL dithiothreitol (DTT) in 100 mM Tris-HCl (pH 8.5) and 5 μL of 50 mg/mL iodoacetamide (IAA) in 100 mM Tris-HCl (pH 8.5) as described [30]. The S-carbamoylmethylated samples were diluted with 56 μL of 100 mM Tris-HCl (pH 8.5), and treated with 2 μL of 0.5 μg/μL lysyl endopeptidase (Lys-C, Wako Pure Chemical Industries) at 25°C for 3 h. Subsequently, samples were digested with 2 μL of 0.5 μg/μL trypsin (Promega) at 37°C for 16 h.

### Sample clean-up for LC-MS/MS analysis

For LC-MS/MS analysis with micro LC, 1000 fmol of stable isotope-labeled peptides was added to half the total volume of sample digests. Sample clean-up was performed manually with GL-tip GC (GL Science Inc., Tokyo, Japan), according to the manufacturer’s protocol. To clean up 192 samples simultaneously, a 96-well centrifuge adapter for the 200 μL tip (GL Science Inc., Tokyo, Japan) coupled with a PT-21 swing rotor (Kubota Corporation, Tokyo, Japan) was used. The resultant eluates were evaporated on a hot plate at 50°C, and residues were reconstituted in 100 μL of 0.1% formic acid/water. One-tenth volume of the samples, corresponding to 0.1 μL plasma, were subjected to LC-MS/MS analysis. For LC-MS/MS analysis with HPLC, samples were not desalted. Forty microliter aliquots of sample digests were spiked with 750 fmol of stable isotope-labeled peptides. Two-thirds (by volume) of the sample was subjected to LC-MS/MS with HPLC analysis.

### Multiplexed SRM/MRM analysis with LC-MS/MS

LC-MS/MS analysis was performed with an electrospray ionization-triple quadrupole mass spectrometer (QTRAP5500; AB SCIEX, Framingham, MA) coupled with a micro LC system (expert microLC 200; Eksigent Technologies, Dublin, CA, USA) or an HPLC system (Agilent 1200 systems; Agilent Technologies, Santa Clara, CA). Micro LC was performed with a C18 column (HALO C18 2.7 μm, 0.5 × 50 mm, Eksigent Technologies). Mobile phases A and B consisted of 0.1% formic acid in water and 0.1% formic acid in acetonitrile, respectively. The peptides were separated and eluted from the column with a linear gradient sequence (10 min run time; flow rate of 20 μL/min for 0–7.5 min and 50 μL/min for 8–10 min) as follows (A: B): 99: 1 for 0–2 min after injection, 80: 20 at 6 min, 0: 100 at 6.5 and up to 7.5 min, 99: 1 at 8 min and up to 10 min. HPLC was performed with C18 capillary columns (Waters XBridge^TM^ BEH130 C18, 1.0 mmID x 100 mm, 3.5 μm particles; Waters, Milford, MA). Mobile phases A and B were the same as for micro LC. The sequence (130 min run time at a flow rate of 50 μL/min) was as follows (A: B): 99: 1 for 5 min after injection, 40: 60 at 65 min, 0: 100 at 66 min and up to 68 min, 99: 1 at 70 min and up to 130 min. The mass spectrometer was set up to run in multiplexed SRM/MRM mode for peptide detection, using a 10 msec per transition. Each target peptide and corresponding isotope-labeled peptide were measured (S1 Table). Unless otherwise stated, LC-MS/MS analysis was performed with micro LC in this report.

Serial dilutions of target peptides (5, 25, 50, 250, 500, 2500 and 5000 fmol) spiked with 1000 fmol of labeled peptides were prepared to obtain standard curves of LC-MS/MS analysis. One-tenth volume of sample was subjected to LC-MS/MS analysis. Systematic error due to incomplete isotope labeling of peptides was corrected by use of a standard curve. In addition, systematic error due to interference of overlapping precursor ions was minimized by use of average values from 4 SRM/MRM transitions as the quantitative values; outliers, as determined by Smirnov-Grubbs’ outlier test (p<0.05), were excluded from calculation of the average values. Ion suppression effects were compensated by using target-to-isotope labeled peptide peak area ratio. Single LC-MS/MS analysis was performed for each clinical plasma sample. To illustrate the reliability of the LC-MS/MS analysis, the average and median %CV values of 6 peptides in the early-stage set and all-stage set are shown in supplemental S3 Table. Most of the %CV values were within 20% (20.8% at maximum) in both sets.

The ion counts in the chromatograms were determined by using an auto analysis system established our laboratory [30]. Peak identification was based on the fact that the unlabeled peptides showed identical retention times to the corresponding labeled peptides, and the peak area counts were greater than 1000 and 5000 counts for LC-MS/MS with micro LC analysis and HPLC analysis, respectively. Raw data files of LC-MS/MS analysis have been deposited in PeptideAtlas (http://www.peptideatlas.org/, Identifier: PASS00756).

### Statistical Analysis

F-test was performed to assess the equality of variance between two groups. Student’s t-test (equal variance) or Welch’s test (unequal variance) was used to determine the statistical significance of differences between two groups. Receiver operating characteristics (ROC) analysis was performed using PRISM6 (Graph Pad, La Jolla, CA), Ekuseru-Toukei 2015 (Social Survey Research Information Co., Ltd., Tokyo, Japan) and R project (http://www.R-project.org). ROC curves were created by plotting the sensitivity (y-axis) and 1-specificity (x-axis) at various thresholds. The optimal thresholds for biomarker candidates were determined as the points with minimum distance from sensitivity and 1-specificity in the ROC curves. The thresholds for currently available markers, CA19-9, DUPAN-2 and CEA, are the standard values for clinical diagnosis. ROC analysis was performed for disease samples against healthy control. For diseases other than IDACP, ROC analysis was also performed against IDACP.

The statistical significance of AUC was determined using Ekuseru-Toukei 2015. Comparison of diagnostic performances between two markers were also performed using Ekuseru-Toukei 2015. A value of p<0.05 was considered as statistically significant. Orthogonal partial least squares discriminant analysis (OPLS-DA) was performed using SIMCA software, version 13 (Umetrics AB, Umea, Sweden). Multivariate logistic regression was performed by using R project.

## Results

### RPPA-based biomarker screening for IDACP

Among 260 upregulated genes in IDACP cells reported by Nakamura *et al*. [13], the biological function of 197 genes are known according to GenBank. The antibodies for 130 proteins among those encoded by the 197 genes were obtained from Abnova (S2 Table). RPPAs were performed for 362 plasma samples listed in Table 1 (set for RPPAs), and ROC analysis of the data for healthy controls (n = 106) and IDACP patients (n = 164) showed that the values of AUC for 23 proteins were more than 0.8 (Table 2). Therefore, we selected these 23 proteins as plasma biomarker candidates for IDACP patients.

**Table 2:** Lists of proteins with AUC≧0.8 in RPPAs.

Protein name	Uniprot accession No.	Plate Number	AUC in RPPAs
AK3L1	P27144	2	0.801
ANXA6	P08133	4	0.845
AP3B1	O00203	3	0.821
ATP6S1	Q15904	3	0.814
C2	P06681	3	0.883
CD82	P27701	4	0.821
CKS1B	Q5T178	2	0.877
CKS2	P33552	4	0.864
CSPG2	P13611	4	0.835
CYCS	P99999	3	0.826
EVI1	Q03112	3	0.846
HMGB2	P26583	4	0.867
HYOU1	Q9Y4L1	2	0.813
IGFBP2	P18065	2	0.850
MMP9	P14780	3	0.856
MST4	Q9P289	2	0.802
MYBL2	P10244	4	0.815
PI3	P19957	4	0.832
PPM1B	O75688	3	0.853
RNASE1	P07998	2	0.805
RNASET2	O00584	2	0.858
STMN1	P16949	3	0.803
VRK2	Q86Y07	2	0.860

The protein list of AUC≧0.8 in RPPAs. Plate number indicates the plasma dilution times in RPPAs for each protein as follows (1; 8 times, 2; 16 times, 3; 32 times, 4: 64 times).

### LC-MS/MS-based High-Throughput Quantification Method for IDACP Biomarker Candidates

Target trypsin peptides (S1 Table) for the 23 proteins identified by RPPAs were selected based on the *in silico* peptide selection criteria described in our previous reports [25,26]. To identify the target peptides detectable in plasma by LC-MS/MS with HPLC, the tryptic digests of plasma from 5 IDACP patients were measured by SRM/MRM with spiking the stable isotope labeled peptides as internal standards. As a result, Complement C2 (C2b) and insulin-like growth factor-binding protein 2 (IGFBP2) were detected in more than 3 SRM/MRM transitions out of 4 (data not shown), among the 23 peptides. As C2 is split into C2a and C2b during the activation pathway [36], we also synthesized target peptides for C2a (S1 Table). IGFBP2 is a family which is consists of six structurally similar proteins (IGFBP1-6) [37]. Therefore, there is a possibility that these proteins would also be good markers for IDACP. Among them, IGFBP1 and 3 were shown to be associated with cancer risk or outcome [38]. Hence, target peptides for IGFBP1 and IGFBP3 were also synthesized. Target peptides for C-reactive protein (CRP) and adiponectin were synthesized because these proteins are also related to cancer risk diseases, such as inflammation and obesity [39]. Standard curves for the peptides except for IGFBP1, which was not detected in plasma from IDACP patients, were prepared by LC-MS/MS (10 min/run). The quantification range for all peptides was from 0.5 to 500 fmol, with an r^2^ value of more than 0.99 (S1 Fig).

To achieve high-throughput and reproducible sample preparation, we have developed an automated robotic sample preparation system including protein reduction, alkylation and digestion (STARplus). The robot was developed to prepare 192 samples (96-well plate×2) in parallel within 24 h. The combination of automated sample preparation (1372 samples/week), LC-MS/MS with micro LC (1008 samples/week) and an auto analysis system developed our laboratory (more than 1000 samples/week) could achieve about 1000 samples analysis within a week. (S2 Fig).

**Fig 1:**
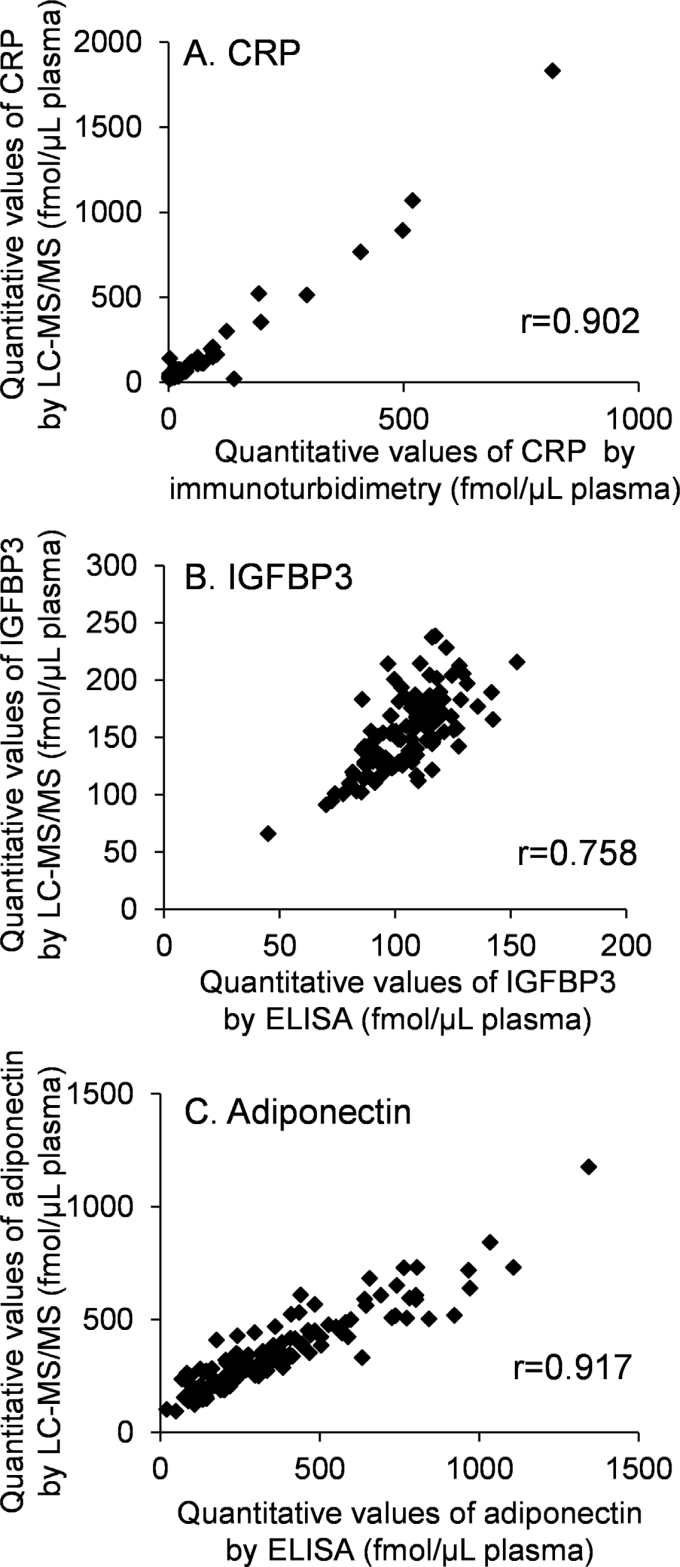
Comparison of quantitative values obtained by LC-MS/MS and antibody-based absolute quantification (immunoturbidimetry and ELISA). (A) CRP was measured by LC-MS/MS and immunoturbidimetry (n = 125). (B and C) IGFBP3 and adiponectin were measured by LC-MS/MS and ELISA (n = 125). The units of the quantitative values determined by immunoturbidimetry and ELISA were converted from gram to mol using the appropriate molecular weight based on the amino acid sequences described in uniprot/swiss-prot; CRP, 24.5 kDa; IGFBP3, 28.8 kDa; adiponectin, 24.5 kDa.

**Fig 2:**
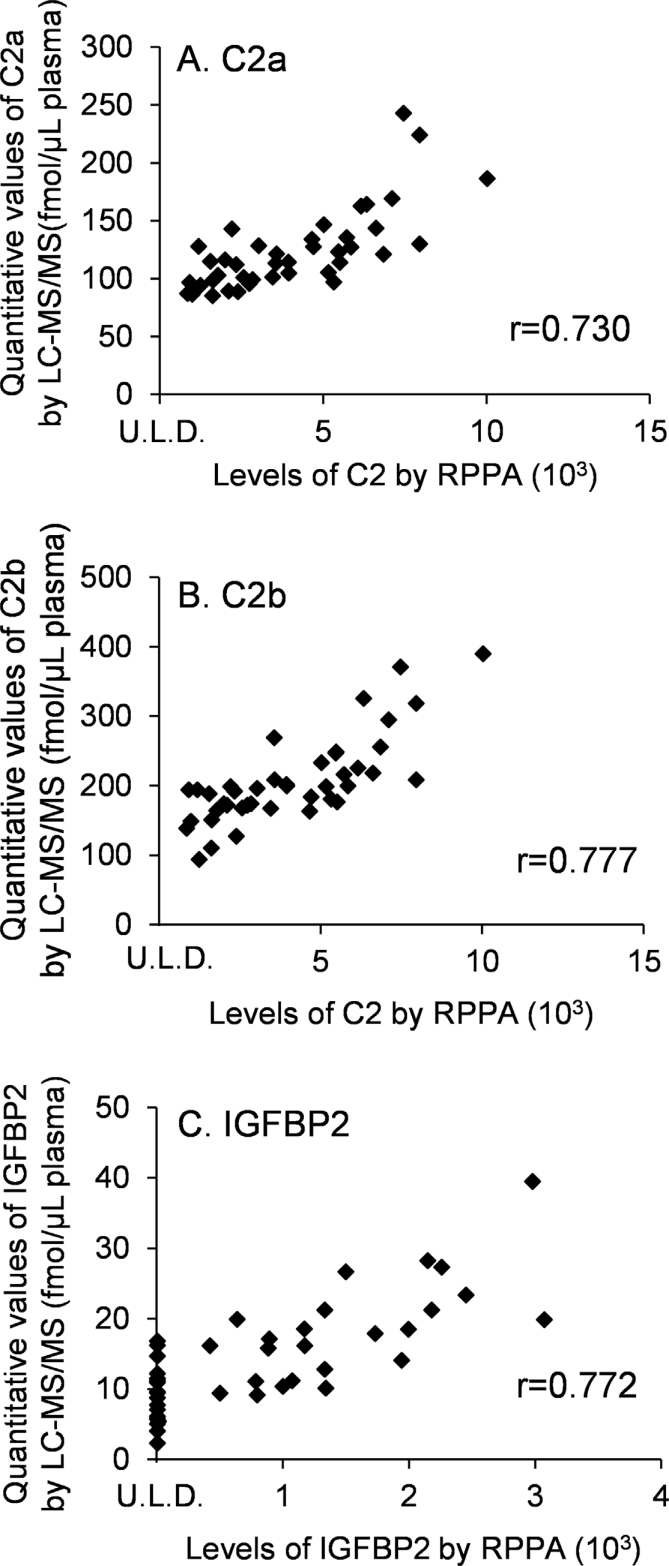
Comparison of the quantitative values obtained by LC-MS/MS and RPPAs. C2 and IGFBP2 were measured by both LC-MS/MS and RPPAs (n = 42). U.L.D., under the limit of detection.

To validate the reproducibility of the developed workflow, the same standard human serum samples (n = 96) were prepared by STARplus and quantified by LC-MS/MS. The quantitative values for each protein were determined as the average values from 4 SRM/MRM transitions shown in S1 Table. The values of % CV (n = 96) of quantitative values were within 10% except for adiponectin, indicating that this workflow has good reproducibility (Table 3). The quantitative values of CRP, IGFBP3 and adiponectin determined by the developed workflow and antibody-based absolute quantification methods (immunoturbidimetry or ELISA) were compared (Fig 1). The values of the Pearson product-moment correlation coefficients (r) of CRP and adiponectin, but not IGFBP3, were more than 0.9, indicating that the quantitative values of CRP and adiponectin obtained by antibody-based absolute quantification method and the developed method are in good agreement. The quantitative values of C2 and IGFBP2 determined by the developed workflow and RPPAs were also compared (Fig 2), and the values of r of C2a, C2b and IGFBP2 were less than 0.8.

**Table 3:** Reproducibility of the Quantitative Values.

Protein Name	Quantitative value (fmol / μL serum)	CV (%)
C2a	189 ± 9	4.68
C2b	195 ± 8	4.31
CRP	40.9 ± 3.8	9.21
IGFBP2	10.2 ± 0.8	8.21
IGFBP3	113 ± 7	6.00
Adiponectin	116 ± 18	15.2

Two microliters of standard serum (n = 96) was digested with Lys-C and trypsin by using the auto preparation machine. The digested samples spiked with isotope-labeled peptides were analyzed by LC-MS/MS. Mean ±SD and CV(%) of the quantitative values are shown.

### Validation of Biomarker Candidates for IDACP by LC-MS/MS in Plasma of the Early-Stage Set

**Fig 3:**
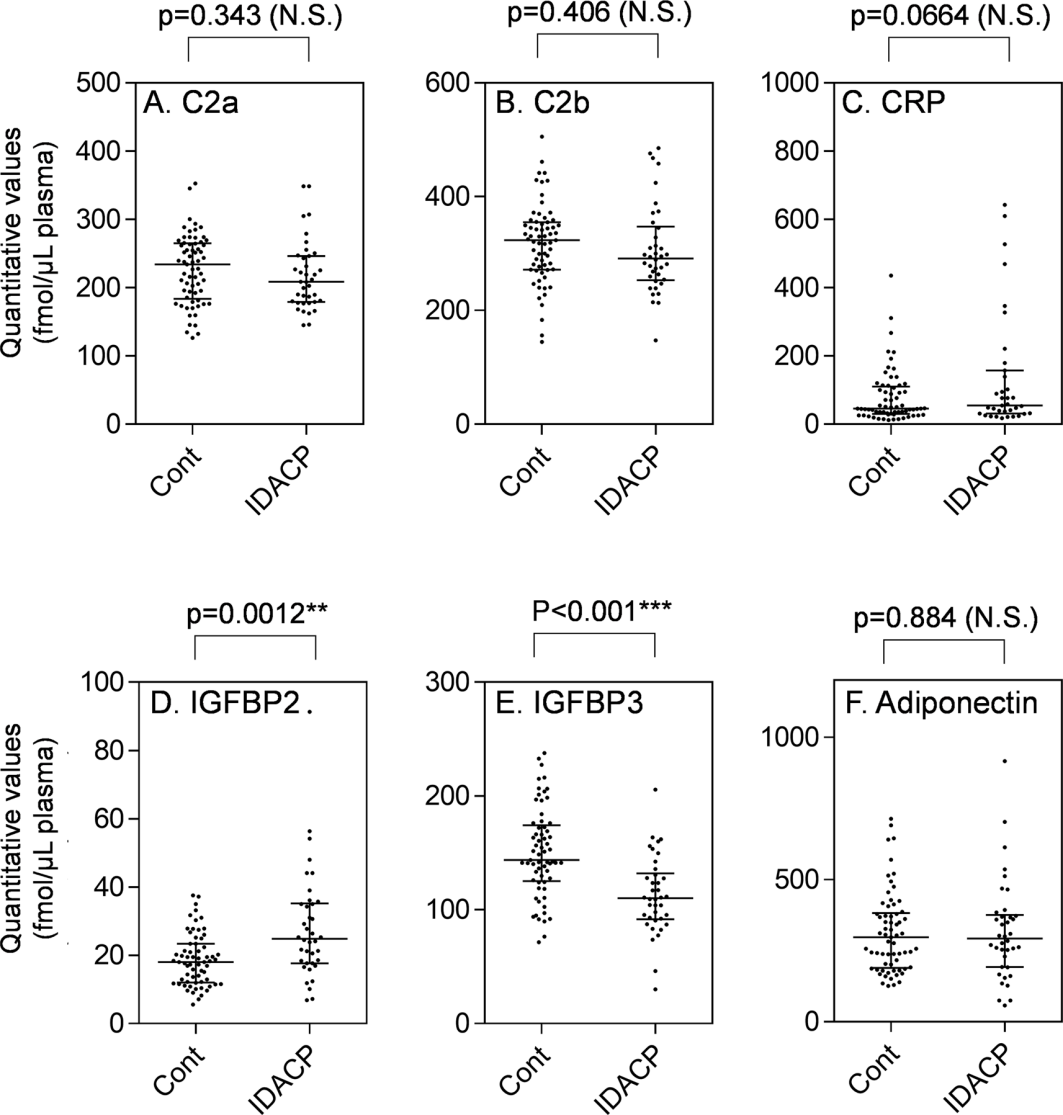
Dot plot showing the differences of IDACP marker candidate between healthy controls (n = 65) and early-stage IDACP (n = 38) in the early-stage set. Each dot represents the protein level in an individual sample, and lines represent median and quartiles. N.S., no significant difference. Cont, healthy controls.

Five proteins (6 peptides) were quantified in the early-stage set, which is composed of 38 stage I or II IDACP patients, and 65 healthy controls (Table 1, early-stage set) in order to validate these proteins as early-stage IDACP biomarkers. As shown in Fig 3, the quantitative values of IGFBP2 and IGFBP3 of patients were significantly greater and lower than those of the controls, respectively. There were no significant differences for the other biomarker candidates between patients and controls, indicating that these proteins are not useful biomarkers for detection of early-stage IDACP. Next, ROC analysis was performed to evaluate the diagnostic performance of IGFBP2 and IGFBP3 as biomarkers and to calculate the thresholds (Table 4). ROC curve analysis revealed that quantitative values of IGFBP2 and IGFBP3 gave AUC values of 0.706 (95% CI, 0.597–0.817) and 0.766 (95% CI, 0.672–0.856), respectively. These AUC values are lower than those of CA19-9 and DUPAN-2, but the sensitivities of IGFBP2 and IGFBP3 are greater than those of CA19-9 and DUPAN-2.

**Table 4:** Receiver Operating Characteristics (ROC) of Markers for IDACP (early-stage set).

Name	AUC (95% CI)	Threshold	Sensitivity (%)	Specificity (%)	Odds ratio
CA19-9	0.836 (0.747–0.925)	>37 U/mL	60.5	92.3	18.4
DUPAN-2	0.835 (0.743–0.927)	>150 U/mL	47.4	96.9	28.4
CEA	0.547 (0.434–0.661)	>2.5 ng/mL	31.6	63.1	0.788
IGFBP2	0.706 (0.597–0.814)	>20.4 fmol/μL plasma	68.4	67.7	4.54
IGFBP3	0.766 (0.672–0.856)	<132 fmol/μL plasma	76.3	70.7	7.80

Thresholds for IGFBP2 and IGFBP3 were determined as the points with minimum distance from 100% sensitivity and 100–0%specificity in the ROC curve for IDACP (n = 38) and healthy controls (n = 65) in the early-stage set. The thresholds for CA19-9, DUPAN-2 and CEA are the standard values for clinical diagnosis. AUC is the area under the ROC curve, and the range of 95% CI is shown. Odds ratio was calculated as %sensitivity × %specificity / (100-%sensitivity) × (100-%specificity).

To assess compensatory ability for CA19-9, the values of each marker in CA19-9-negative patients (<37 U/mL) are shown in Table 5. Among 15 CA19-9-negative patients, IGFBP2 and IGFBP3 were positive in 8 and 10 patients, whereas only 3 patients were positive for both DUPAN-2 and CEA. OPLS-DA was performed by SIMCA software to identify the optimum markers to separate controls and patients. The variable importance of projection (VIP) scores for CA19-9, DUPAN-2, CEA, C2a, C2b, CRP, IGFBP2, IGFBP3 and adiponectin were 1.61, 1.63, 0.207, 0.347, 0.304, 0.901, 1.39, 1.62 and 0.0537, respectively. The VIP scores for CA19-9, DUPAN-2, IGFBP2 and IGFBP3 are greater than 1, indicating that these markers can separate controls and patients. These results indicate that IGFBP2 and IGFBP3 may have compensatory ability for CA19-9.

**Table 5:** Levels of Markers in Plasma of CA19-9-Negative IDACP Patients.

Stage	CA19-9 (U/mL)	CEA (ng/mL)	DUPAN-2 (U/mL)	IGFBP2 (fmol/μL plasma)	IGFBP3 (fmol/μL plasma)
I	8.1	1.9	25	44.1*	164
II	11.7	1.3	25	25.3*	104*
II	30.5	1.3	36	16.6	128*
II	11.5	1	25	10.1	111*
II	28.9	1.6	25	6.84	153
II	17.1	5.6*	25	39.0*	98.0*
II	25.9	2.2	42	24.1*	111*
II	26.9	2.1	88	20.6*	128*
II	1	2.2	300*	18.6	162
II	8.4	3.1*	25	17.7	142
II	12.6	0.9	25	11.8	117*
II	5.7	1.1	25	12.4	87.3*
II	1	2	1300*	34.3*	150
II	31.8	1.5	25	25.3*	92.2*
II	15.9	2.9*	1600*	21.2*	73.7*

Levels of markers in plasma of CA19-9-negative IDACP patients are shown. The values shown in bold with asterisks are above and below the thresholds for IGFBP2 and IGFBP3 shown in Table 4, respectively.

### Combination Diagnosis using CA19-9, IGFBP2 and IGFBP3 with Multivariate Logistic Regression Model

To assess the value of combination diagnosis using CA19-9, IGFBP2 and IGFBP3, these markers were subjected to multivariate logistic regression analysis by R project. The developed predictive model is shown by Eq 1. The logistic regression model for CA19-9 was also obtained as Eq 2, for comparison with Eq 1.

Probability=1/(1+exp(−0.652+0.0299×CA19-9+0.0390×IGFBP2−0.0245×IGFBP3))) (Eq 1)

Probability=1/(1+exp(−(2.57+0.0589×CA19-9))) (Eq 2)

**Fig 4:**
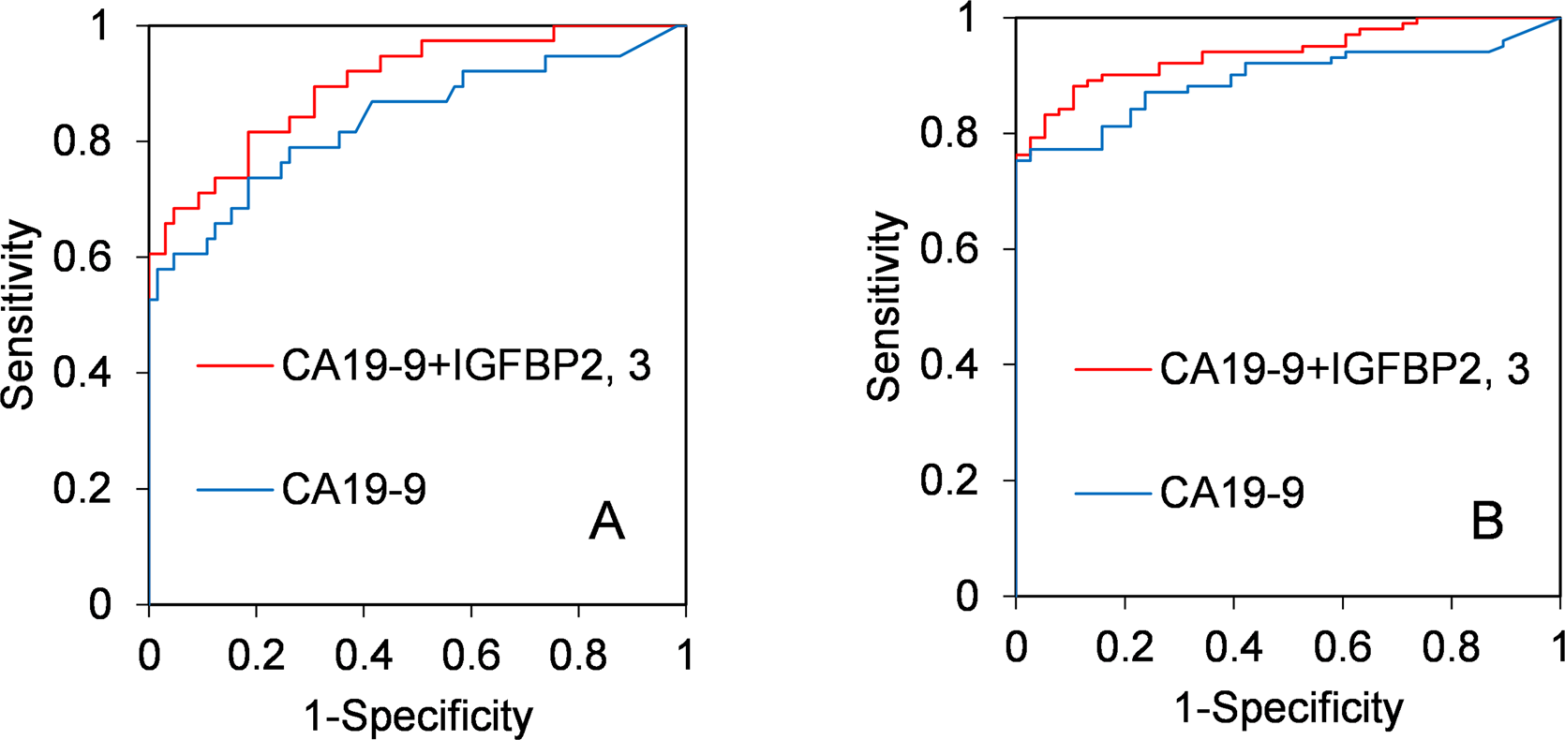
ROC curves of multivariate logistic regression. (A) Multivariate logistic regression formulae of CA19-9+IGFBP2, 3 (AUC, 900; 95% CI, 0.837–0.962) and CA19-9 only (AUC, 836; 95% CI, 0.746–0.926) were produced from the quantitative values of the early-stage set, and ROC analysis was performed. (B) The same formulae of CA19-9+IGFBP2, 3 (AUC, 940; 95% CI, 0.903–0.976) and CA19-9 only (AUC, 0.894; 95% CI, 0.842–0.946) were applied to the quantitative values of the all-stage set, and ROC analysis was performed.

This model (Eq 1) discriminated well between controls and patients in the early-stage set (AUC, 0.900; 95% CI, 0.837–0.962) (Fig 4A). To compare the diagnostic performance between Eq 1 and Eq 2, the difference between AUC (the value of AUC from Eq 1 –the value of AUC from E 2) was calculated (difference between AUC, 0.064; 95% CI, 0.001–0.127; p = 0.0477) based on a previous report [40]. The significant p-value (p<0.05) suggests that the diagnostic performance of Eq 1 is better than that of Eq 2.

### Validation of Logistic Model by Using the All-Stage Set

**Fig 5:**
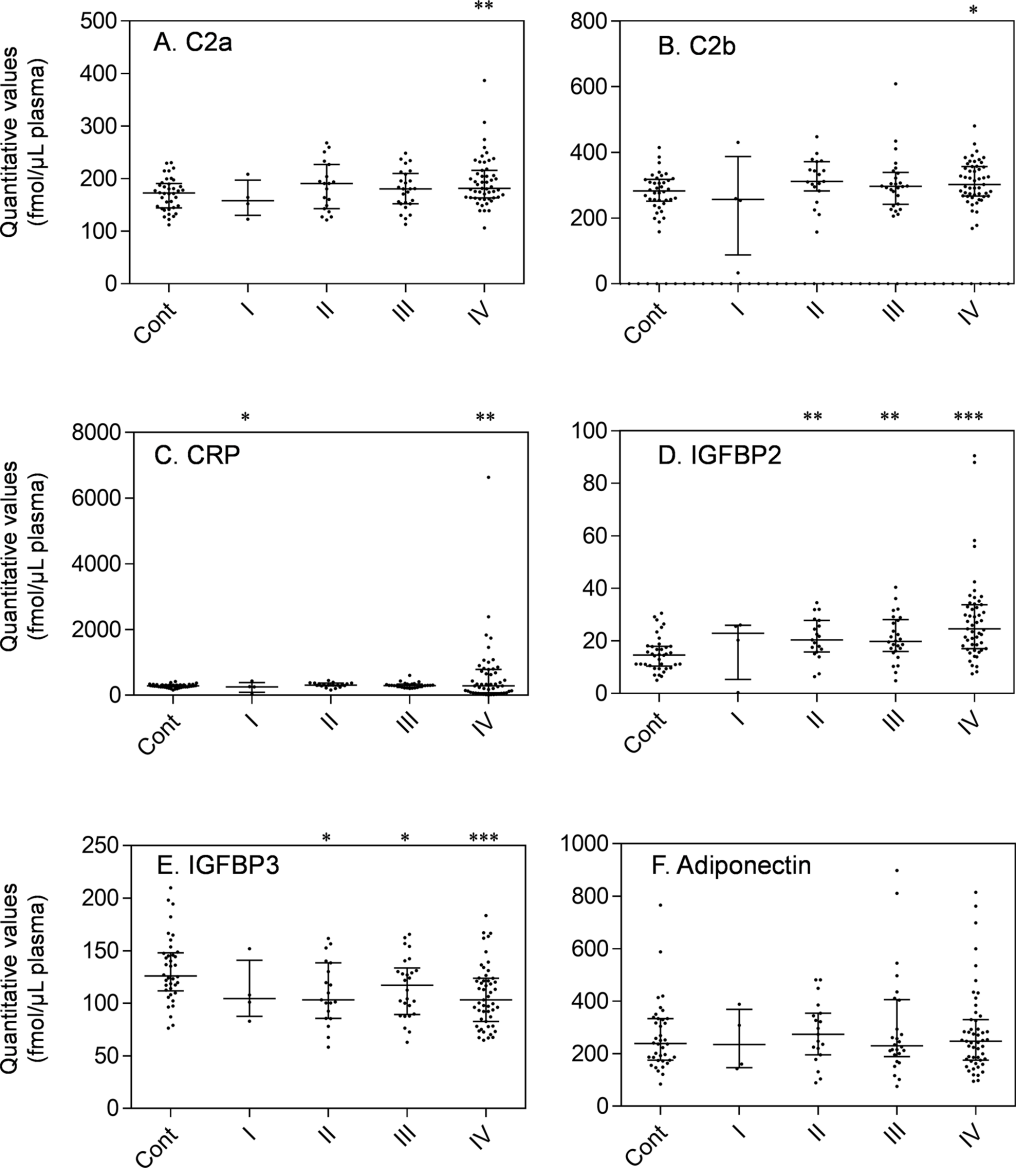
Dot plot showing the plasma levels of each IDACP biomarker candidate in controls (n = 38) and patients with stage I (n = 4), II (n = 19), III (n = 26) and IV (n = 51) in the all-stage set. Each dot represents the protein level of an individual sample, and lines represent median and quartiles. *, p<0.05; **, p<0.01; ***, p<0.001. Healthy controls, cont.

The other set (all-stage set) of controls (n = 38) and stage I-IV IDACP patients (n = 101) shown in Table 1, which did not overlap with early-stage set, was quantified to evaluate the markers in more detail. As shown in Fig 5, the quantitative values of IGFBP2 and IGFBP3 were significantly increased and decreased, respectively, from those of stage II IDACP patients, which correspond to the early-stage set (Fig 3). The quantitative values of C2a, C2b and CRP were significantly elevated in stage IV IDACP patients, suggesting that these markers may only be useful for diagnosis of late-stage IDACP. A dot plot of all proteins in the all-stage set is shown in S3 Fig.

To evaluate the diagnostic performance of eqn1, ROC analysis of Eq 1 was carried out using the quantitative values of CA19-9, IGFBP2 and IGFBP3 in the all-stage set. As shown in Fig 4B, this model (Eq 1) discriminated well between controls and patients (AUC, 0.940; 95% CI, 0.903–0.976). To compare the diagnostic performance between Eq 1 (CA19-9+IGFBP2+IGFBP3) and Eq 2 (CA19-9) in the all-stage set, the difference between AUC (AUC of Eq 1-AUC of Eq 2) was also calculated (difference between AUC, 0.046; 95% CI, 0.006–0.085; p = 0.0226). The significant p-value (p<0.05) validates the performance of eqn1 for pancreatic cancer diagnosis with samples other than those used for development of the model.

### IGFBP2, 3 Levels for Diagnosing Diseases other than IDACP

We also performed ROC analysis of IGFBP2, IGFBP3, CA19-9, DUPAN-2 and Eq 1 in patients who would be considered at risk for pancreatic malignancy due to other diseases, such as mucinous cystic neoplasms (MCNs), intraductal papillary mucinous neoplasms (IPMNs), endocrine neoplasms, chronic pancreatitis and other conditions shown in Table 1 (all-stage set).We performed ROC analysis of these diseases against healthy controls and IDACP patients (Table 6). Significant AUC values of IGFBP2 were observed with IPMNs, endocrine neoplasms and chronic pancreatitis against healthy controls (Table 6). The AUC values of other markers showed no significance against healthy controls. On the other hand, the markers other than IGFBP2 exhibited significant AUC values discriminating most pancreatic diseases from IDACP. Eq 1 and CA19-9 discriminated all pancreatic diseases from IDACP with similar AUC values of more than 0.8 (Table 6 and S4 Fig).

**Table 6:** IGFBP2, 3, CA19-9 and DUPAN-2 as markers in patients with pancreatic diseases other than IDACP.

		N	AUC (95% CI)				
			IGFBP2	IGFBP3	Eq 1	CA19-9	DUPAN2
MCNs	vs healthy control	5	0.518 (0.220–0.817)	0.674 (0.410–0.937)	0.511 (0.130–0.891)	0.511 (0.189–0.832)	0.742 (0.444–1.04)
	vs IDACP		0.711 (0.413–1.01)	0.834* (0.660–1.01)	0.802* (0.547–1.06)	0.800* (0.612–0.988)	0.515 (0.199–0.831)
IPMNs	vs healthy control	25	0.764* (0.646–0.882)	0.568 (0.423–0.714)	0.619 (0.471–0.757)	0.524 (0.374–0.675)	0.517 (0.372–0.663)
	vs IDACP		0.531 (0.404–0.657)	0.638* (0.525–0.751)	0.914* (0.863–0.965)	0.888* (0.832–0.944)	0.868* (0.806–0.930)
Endocrine neoplasms	vs healthy control	11	0.788* (0.646–0.930)	0.531 (0.329–0.734)	0.538 (0.357–0.720)	0.507 (0.309–0.706)	0.526 (0.320–0.732)
	vs IDACP		0.520 (0.363–0.678)	0.712* (0.551–0.873)	0.933* (0.879–0.988)	0.889* (0.825–0.953)	0.843* (0.755–0.931)
Chronic pancreas	vs healthy control	3	0.860* (0.749–0.971)	0.579 (0.242–0.916)	0.526 (0.273–0.779)	0.623 (0.463–0.782)	0.632 (0.364–0.899)
	vs IDACP		0.526 (0.380–0.673)	0.762* (0.545–0.980)	0.957* (0.907–1.01)	0.934* (0.886–0.982)	0.911* (0.835–0.986)
Others	vs healthy control	6	0.561 (0.308–0.815)	0.667 (0.499–0.834)	0.588 (0.378–0.800)	0.588 (0.316–0.859)	0.568 (0.290–0.846)
	vs IDACP		0.715* (0.514–0.915)	0.584 (0.435–0.734)	0.893* (0.789–0.996)	0.889* (0.787–0.990)	0.790* (0.632–0.948)

AUC and 95% CI for pancreatic diseases other than IDACP against healthy controls and IDACP are shown. Statistically significant AUC values (p<0.05) are shown in bold with asterisks.

We also performed ROC analysis of IGFBP2, IGFBP3, CA19-9, DUPAN-2 and Eq 1 in patients with malignancies other than IDACP shown in Table 1 (all-stage set) against healthy controls and IDACP patients (Table 7). Significant AUC values of IGFBP2 were observed in gastric cancer, cholangiocarcinoma, hepatocellular carcinoma, colon cancer and duodenal cancer (Table 7) against healthy controls. The levels of IGFBP2 determined by RPPA were also increased in gastric cancer, cholangiocarcinoma, hepatocellular carcinoma, and colon cancer compared to healthy controls (S5 Fig), which corresponded to the result of LC-MS/MS. Significant AUC of IGFBP3 against healthy controls was observed in cholangiocarcinoma and hepatocellular carcinoma. However, the AUC values of IGFBP2 and 3 between most malignancies and IDACP were not so high (AUC < 0.8), except in the case of IGFBP3 for hepatocellular carcinoma, suggesting that these proteins have limited specificity for IDACP diagnosis. IGFBP3 discriminated hepatocellular carcinoma from either healthy controls or IDACP with an AUC of more than 0.8. CA19-9 exhibited a high AUC (>0.8) for IDACP versus esophagus cancer, gastric cancer, hepatocellular carcinoma and colon cancer. Eq 1 also showed a high AUC (>0.784) in the same comparison between IDACP and other malignancies, and, in addition, Eq 1 gave a high AUC (>0.8) for healthy controls versus cholangiocarcinoma, healthy controls versus hepatocellular carcinoma, and IDACP versus duodenal cancer. Therefore, Eq 1 appears to effectively combine the marker abilities of IGFBP2, 3 and CA19-9.

**Table 7:** IGFBP2, 3, CA19-9 and DUPAN-2 as markers in patients with malignancies other than IDACP.

			AUC (95% CI)				
		N	IGFBP2	IGFBP3	Eq 1	CA19-9	DUPAN-2
Esophagus cancer	vs healthy control	10	0.697 (0.489–0.906)	0.624 (0.370–0.878)	0.684 (0.468–0.901)	0.570 (0.380–0.759)	0.511 (0.299–0.722)
	vs IDACP		0.517 (0.279–0.755)	0.515 (0.257–0.773)	0.868* (0.783–0.954)	0.883* (0.817–0.950)	0.830* (0.708–0.952)
Gastric cancer	vs healthy control	119	0.721* (0.630–0.812)	0.555 (0.458–0.651)	0.624* (0.530–0.718)	0.535 (0.432–0.638)	0.505 (0.400–0.610)
	vs IDACP		0.559 (0.483–0.635)	0.618* (0.544–0.692)	0.875* (0.828–0.922)	0.859* (0.806–0.913)	0.845* (0.791–0.900)
Cholangiocarcinoma	vs healthy control	24	0.879* (0.797–0.961)	0.700* (0.558–0.841)	0.833* (0.715–0.952)	0.741* (0.595–0.888)	0.959* (0.915–1.00)
	vs IDACP		0.644* (0.524–0.764)	0.502 (0.369–0.635)	0.670* (0.548–0.793)	0.676* (0.554–0.800)	0.554 (0.441–0.666)
Hepatocellular carcinoma	vs healthy control	12	0.842* (0.731–0.953)	0.945* (0.886–1.00)	0.906* (0.824–0.987)	0.520 (0.322–0.717)	0.724* (0.545–0.902)
	vs IDACP		0.534 (0.395–0.673)	0.800* (0.680–0.919)	0.784* (0.666–0.902)	0.860* (0.766–0.953)	0.727* (0.598–0.856)
Colon cancer	vs healthy control	127	0.709* (0.616–0.802)	0.533 (0.438–0.629)	0.596* (0.501–0.691)	0.540 (0.436–0.645)	0.540 (0.434–0.647)
	vs IDACP		0.579* (0.503–0.654)	0.631* (0.559–0.703)	0.873* (0.826–0.920)	0.863* (0.810–0.915)	0.867* (0.815–0.918)
Duodenal cancer	vs healthy control	10	0.824* (0.692–0.956)	0.613 (0.363–0.864)	0.689 (0.458–0.916)	0.754* (0.592–0.916)	0.716* (0.530–0.901)
	vs IDACP		0.555 (0.387–0.724)	0.531 (0.301–0.760)	0.813* (0.669–0.957)	0.763* (0.619–0.907)	0.703* (0.535–0.872)

AUC and 95% CI for malignancies other than IDACP against healthy controls and IDACP are shown. Statistically significant AUC values (p<0.05) are shown in bold with asterisks.

## Discussion

By using the combination of antibody-based proteomics and LC-MS/MS-based proteomics, we have shown that IGFBP2 and IGFBP3 are increased and decreased, respectively, in plasma of early-stage IDACP. IGFBP2 is also increased in plasma of risk diseases for pancreatic malignancies. We further showed that both IGFBP2 and IGFBP3 have compensatory ability for CA19-9 in the diagnosis of IDACP, and thus can improve diagnostic performance.

The IGFBP family consists of six proteins with similar structure, which bind insulin growth factors (IGFs) and regulate the circulatory half-life of the IGFs [41,42]. For example, the half-lives of free IGFs, IGFs/IGFBP3 complex and IGFs/IGFBP3/acid labile subunit (ALS) complex are a few minutes, 20–30 min and 16 hours or longer, respectively [43]. Most of the circulating pool of IGFs is stabilized by association with IGFBP3 and ALS, and the binding affinity of IGFs to IGFBP3 is decreased when IGFBP 3 undergoes limited proteolysis, resulting in degradation of IGFBP3 and activation of the IGF signal [44]. Since decrease of IGFBP3 was observed from an early stage of IDACP (Figs 3 and 5), and there is no difference in levels between the various IDACP stages (Fig 5), activation of the IGF signal might be involved in cancer progression from an early stage of IDACP.

There is no consistent evidence regarding the association between IGFBP3 level and cancer prognosis [45–47]. The conflicting results may be due to differences in the epitopes of the antibodies used for quantification of IGFBP3 in previous reports, because IGFBP3 has multiple circulating forms (i.e. free, complex and fragmented) and the affinity of antibodies for each of these forms can be different [38]. On the other hand, this is not an issue for LC-MS/MS, as the protein is completely digested with enzymes. For this reason, we believe that clinical diagnosis using the quantitative values of IGFBP3 would be better achieved by means of LC-MS/MS, rather than an antibody-based method.

The level of IGFBP2 is reported to be increased in IDACP patients’ tissues [48,49] and serum [50] at the protein level, in accordance with our results. There is abundant evidence that IGFBP2 plays a role in promotion of various cancers [51]. The interaction of the Arg-Gly-Asp motif in IGFBP2 with integrins typically results in stimulatory effects towards cancer cells [38]. Moreover, nuclear transport of IGFBP2 is reported to be associated with tumorigenic effect by promoting angiogenesis through activation of *VEGF* transcription [52,53]. Therefore, there are plausible mechanisms to account for the decrease and increase of IGFBP3 and IGFBP2, respectively, in IDACP patients.

Evaluation by ROC analysis revealed that the values of AUC of IGFBP2 and IGFBP3 in the early-stage set were lower than those of CA19-9 and DUPAN-2 (Table 4). Furthermore, a cancer biomarker should preferably be released/induced/produced by the cancer and should correlate with tumor burden, increasing with tumor growth. The level of IGFBP2 increased slightly as the clinical stage became more advanced. However, the level of IGFBP3 was decreased in IDACP compared to healthy controls, and its level did not change depending on stage. These results suggest that single use of either IGFBP2 or IGFBP3 would have limited clinical utility as a pancreatic cancer biomarker.

To improve the diagnosis of IDACP, the availability of biomarkers to detect CA19-9-negative IDACP patients is important. As shown in Table 5, 8 patients were positive for IGFBP2 and 10 patients were positive for IGFBP3 out of 15 CA19-9-negative patients, whereas only 3 patients were positive for both DUPAN-2 and CEA. A similar result was obtained from the quantitative values of IGFBP2 and IGFBP3 in the all-stage set (S4 Table). Furthermore, based on multivariate logistic regression model (Eq 1), we found that diagnosis of IDACP using the combination of CA19-9, IGFBP2 and IGFBP3 is significantly more effective than CA19-9 alone (Fig 4). These results suggest that IGFBP2 and IGFBP3 have compensatory ability for CA19-9. The differences in AUC between Eq 1 and CA19-9 were statistically significant, but not large (0.064 in the early-stage set and 0.046 in the all-stage set). The AUC of CA19-9 in the present sets is already high (0.836 and 0.894 in the early-stage and all-stage sets, respectively), so further study with a larger number of clinical samples would be necessary to evaluate whether introducing IGFBP2 and 3 into clinical practice would be justified.

IGFBP2 was also suggested to increase in risk diseases of pancreatic malignancy, such as IPMNs (Table 6). Kim *et al*. recently reported that a six-protein panel had high discriminating power in distinguishing between IPMMs and controls, and their protein panel included IGFBP2 and IGFBP3 [54]. This result supports the idea that these molecules are promising biomarker candidates for IPMNs. However, due to the limited number of chronic pancreatitis samples, further study is necessary to establish whether IGFBP2 is available as a marker to discriminate IDACP from chronic pancreatitis. If patients with high-risk diseases for pancreatic malignancies can be distinguished from healthy controls, pancreatic cancer might be detected at an earlier stage than is currently possible [3].

As shown in Table 7, IGFBP2 or 3 had a high AUC (>0.8) for healthy controls versus cholangiocarcinoma and hepatocellular carcinoma. This result is reasonable because the liver, bile ducts and pancreas have a common embryologic origin [55]. Furthermore, IGFBP2 or 3 exhibited a relatively low AUC (<0.8) for discriminating IDACP from other malignancies, except hepatocellular carcinoma in the case of IGFBP3. These results suggest limited specificity of IGFBP2 and 3 for IDACP. Eq 1 may improve the specificity by combining IGFBP2, IGFBP3 and CA19-9. However, it would be desirable to use other screening methods, such as endoscopic ultrasonography, computed tomography and magnetic resonance imaging, in parallel with these markers to discriminate the disease state of those patients.

Comparison of the quantitative values of CRP, IGFBP3 and adiponectin between LC-MS/MS and antibody-based absolute quantification methods (immunoturbidimetry, ELISA) revealed that values of CRP and adiponectin were well correlated (Fig 1A and 1C). This result suggests that quantification by antibody-based absolute quantification methods and LC-MS/MS gives similar results, even though the absolute values obtained by the two methods were slightly different. The r value of IGFBP3 between ELISA and LC-MS/MS is 0.758 (Fig 1B), which is lower than those of CRP and adiponectin, possibly due to a difference in the measured forms of IGFBP3 (i.e. free, complex and fragmented), as discussed above. The values of r for C2a, C2b and IGFBP2, which were quantified by RPPAs and LC-MS/MS, were in the range from 0.730 to 0.777 (Fig 2). These values are lower than those of CRP and adiponectin. In our research, samples for RPPAs were not fractionated and the antibodies for C2 and IGFBP2 are polyclonal, so non-specific signals may have affected the quantitative values of RPPAs. However, both C2 and IGFBP2, which were identified as biomarkers by RPPA, were upregulated in plasma from at least stage IV patients by LC-MS/MS (Fig 5), suggesting that RPPA is a powerful tool for high-throughput biomarker protein screening. After identifying major biomarker candidates by RPPA, reliable quantification of biomarker proteins performed by LC-MS/MS was an effective means to validate the candidates. It is noteworthy that the present strategy, i.e., the combination of two proteomics methods (RPPA and LC-MS/MS), allowed us to identify useful biomarker proteins from among more than 100 candidates.

We have developed a high-throughput and reliable quantification method for C2a, C2b, CRP, IGFBP2, IGFBP3 and adiponectin by combining automated sample preparation, LC-MS/MS with SRM/MRM analysis and an auto analysis system, and the developed method was used to quantify nearly 600 samples. The most important point in developing a high-throughput quantification method based on LC-MS/MS and automated sample preparation is the reproducibility of the quantitative values. The % CV (n = 96) of quantitative values of 6 peptides in standard human serum was within 10% in all cases, except for adiponectin (Table 3). As the criterion for biological analysis is 15% in the FDA guideline [56], this developed method has sufficient reproducibility. The ELISA method is widely used for biomarker protein quantification, but problems arise when multiple protein quantification is needed and only limited sample volumes are available. On the other hand, LC-MS/MS-based biomarker protein quantification allows multiplex quantification of biomarker proteins by analyzing only 0.1 μL plasma. In this regard, the high-throughput LC-MS/MS analysis would be advantageous for combination diagnosis with multiple markers.

## Conclusion

We have developed a new high-throughput biomarker identification strategy based on the combination of RPPA and LC-MS/MS. Analysis of the quantification results of nearly 600 samples indicated that IGFBP2 and IGFBP3 have compensatory ability for CA19-9 in the diagnosis of IDACP. The results indicate that use of the combination of IGFBP2, IGFBP3 and CA19-9 could enable more reliable diagnosis of early-stage IDACP.

## Supporting Information

S1 Fig Standard curves for IDACP biomarker candidate proteins. Serial dilutions of the unlabeled peptides (0.5, 2.5, 5, 10, 25, 50, 100, 250 and 500 fmol) spiked with 100 fmol of isotope-labeled peptide were analyzed by LC-MS/MS. Each data point represent mean ±SD (n = 11 or 12) of data collected for 4 different SRM/MRM transitions in 3 experiments analyzed on different days.
(TIF)

S2 Fig Workflow of high-throughput SRM/MRM method. Automated trypsin digestion procedures were conducted on 192 samples (96-well microplate × 2) within 24 h (1372 samples / week). As the LC-MS/MS run time is 10 min, 1008 samples could be quantified within a week. The auto analysis system could analyze more than 1000 samples within a week. Thus, in total, about 1000 samples could be analyzed per week.
(TIF)

S3 Fig Dot plot showing the plasma levels of each IDACP biomarker candidate in the all-stage set. Lines represent median and quartiles. Healthy controls, cont; endocrine neoplasms, En; pancreatitis, Pa; esophageal cancer, Es; gastric cancer, Ga; cholangiocarcinoma, Ch; hepatocellular carcinoma, He; colon cancer, Co; duodenal cancer, Du. *, p<0.05 compared to healthy controls; ⁺, p<0.05 compared to IDACP.
(TIF)

S4 Fig ROC curves and dot plot of Eq1 between IDACP and pancreatic diseases. (A) ROC curves of Eq 1 among IDACP, pancreatic diseases and healthy controls. AUC values and 95%CI values were shown in Table 6. (B) Dot plot of probability of IDACP, pancreatic diseases and healthy controls calculated from Eq 1. Lines represent median and quartiles.*, p<0.05 compared to healthy controls; ⁺, p<0.05 compared to IDACP.
(TIF)

S5 Fig Box-and-whisker diagram showing IGFBP2 levels measured using RPPA among patients with diverse diseases (listed in Table 1). Healthy controls, cont; pancreatitis, Pa; hepatocellular carcinoma, He; cholangiocarcinoma, Ch; gastric cancer, Ga; colon cancer, Co.
(TIF)

S1 Table SRM/MRM transitions. The peptides for each target protein for LC-MS/MS analysis were selected by using *in silico* criteria [25,26]. The conditions of SRM/MRM were optimized for high signal intensity following direct injection of peptide solution into the mass spectrometer through a turbo ion spray source. Theoretical *m/z* values of doubly or thirdly charged ions of intact peptides (Q1) were assumed as precursor ions. Four singly or doubly charged fragment ions produced from precursor ions were selected as Q3-1, -2, -3 and -4. Bold letters with asterisks show the stable isotope-labeled amino acid residues (^13^C and ^15^N).
(PDF)

S2 Table List of antibodies for RPPAs. All antibodies were obtained from Abnova.
(PDF)

S3 Table The average and median %CV of early-stage set and all-stage set. The %CV values were obtained based on the 4 SRM/MRM transitions.
(PDF)

S4 Table Levels of markers in plasma of CA19-9-negative IDACP patients (all stages). Levels of markers in plasma of CA19-9-negative IDACP patients (all-stage set) are shown. The values marked in gray are above and below the thresholds for IGFBP2 and IGFBP3 shown in Table 4, respectively.
(PDF)
