# Supplementary figures and images for: Identification of IGFBP2 and IGFBP3 As Compensatory Biomarkers for CA19-9 in Early-Stage Pancreatic Cancer Using a Combination of Antibody-Based and LC-MS/MS-Based Proteomics
Source: PLoS One. 2016 Aug 31;11(8):e0161009. doi: 10.1371/journal.pone.0161009 (PMC5007017; doi:10.1371/journal.pone.0161009)

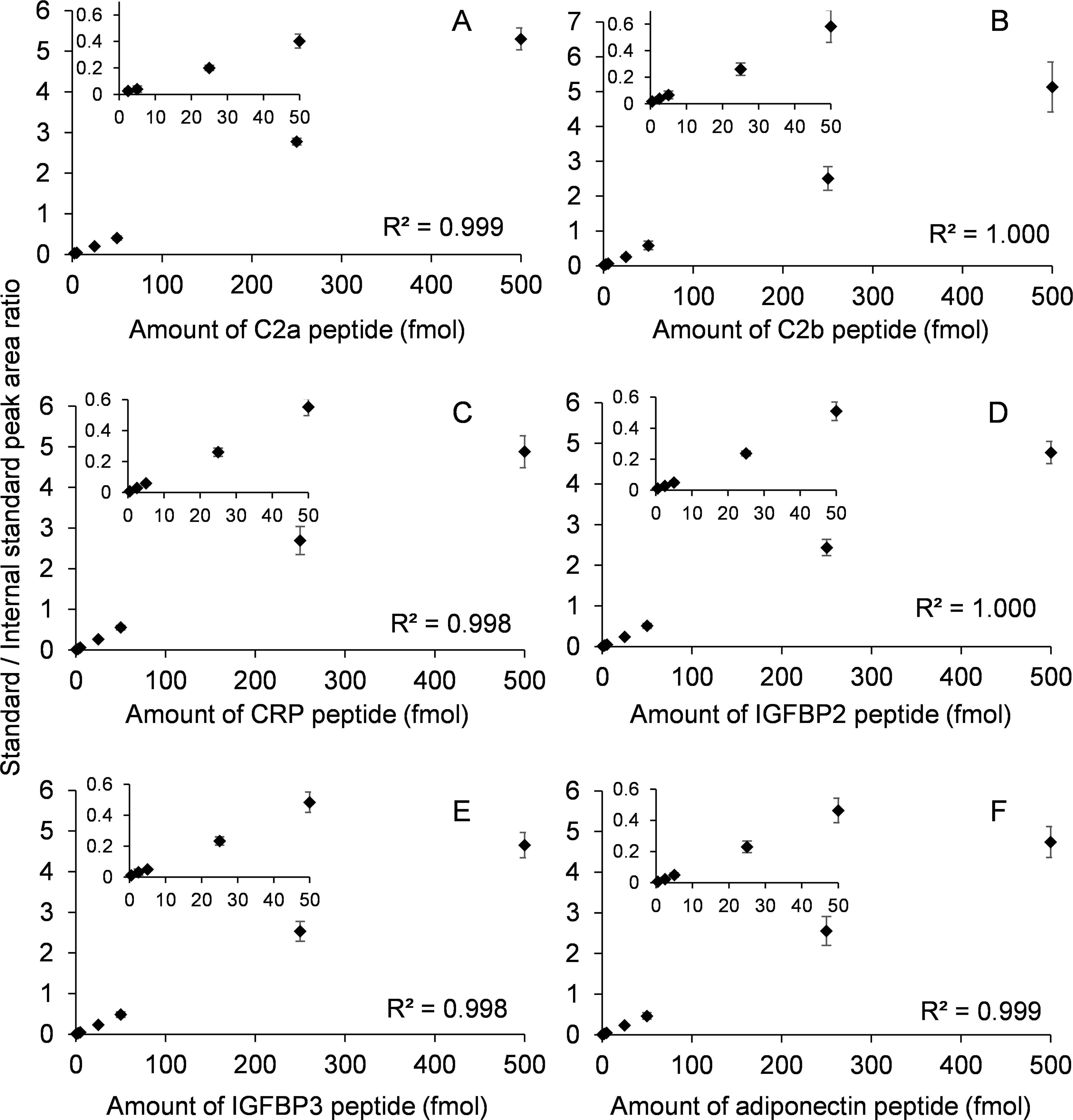

Supplement: S1 Fig — Serial dilutions of the unlabeled peptides (0.5, 2.5, 5, 10, 25, 50, 100, 250 and 500 fmol) spiked with 100 fmol of isotope-labeled peptide were analyzed by LC-MS/MS. Each data point represent mean ±SD (n = 11 or 12) of data collected for 4 different SRM/MRM transitions in 3 experiments analyzed on different days. (TIF) [file pone.0161009.s001.tif]

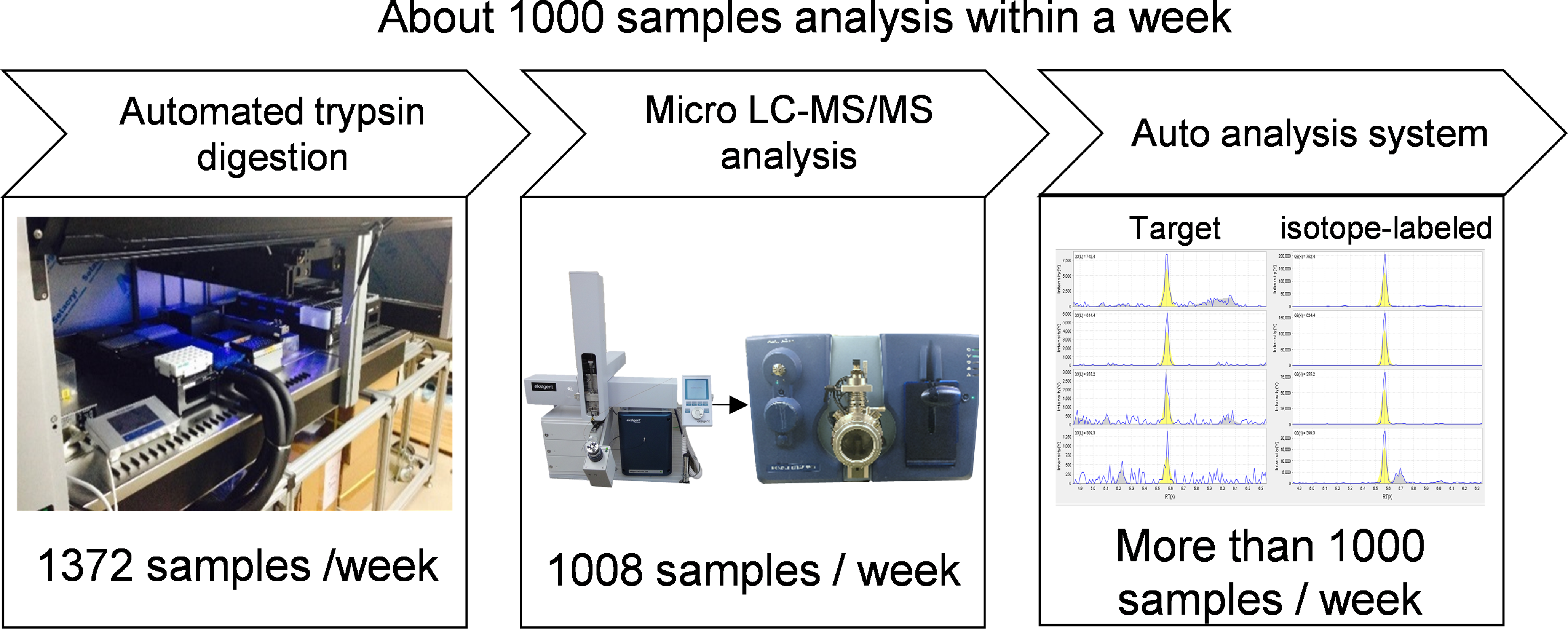

Supplement: S2 Fig — Automated trypsin digestion procedures were conducted on 192 samples (96-well microplate × 2) within 24 h (1372 samples / week). As the LC-MS/MS run time is 10 min, 1008 samples could be quantified within a week. The auto analysis system could analyze more than 1000 samples within a week. Thus, in total, about 1000 samples could be analyzed per week. (TIF) [file pone.0161009.s002.tif]

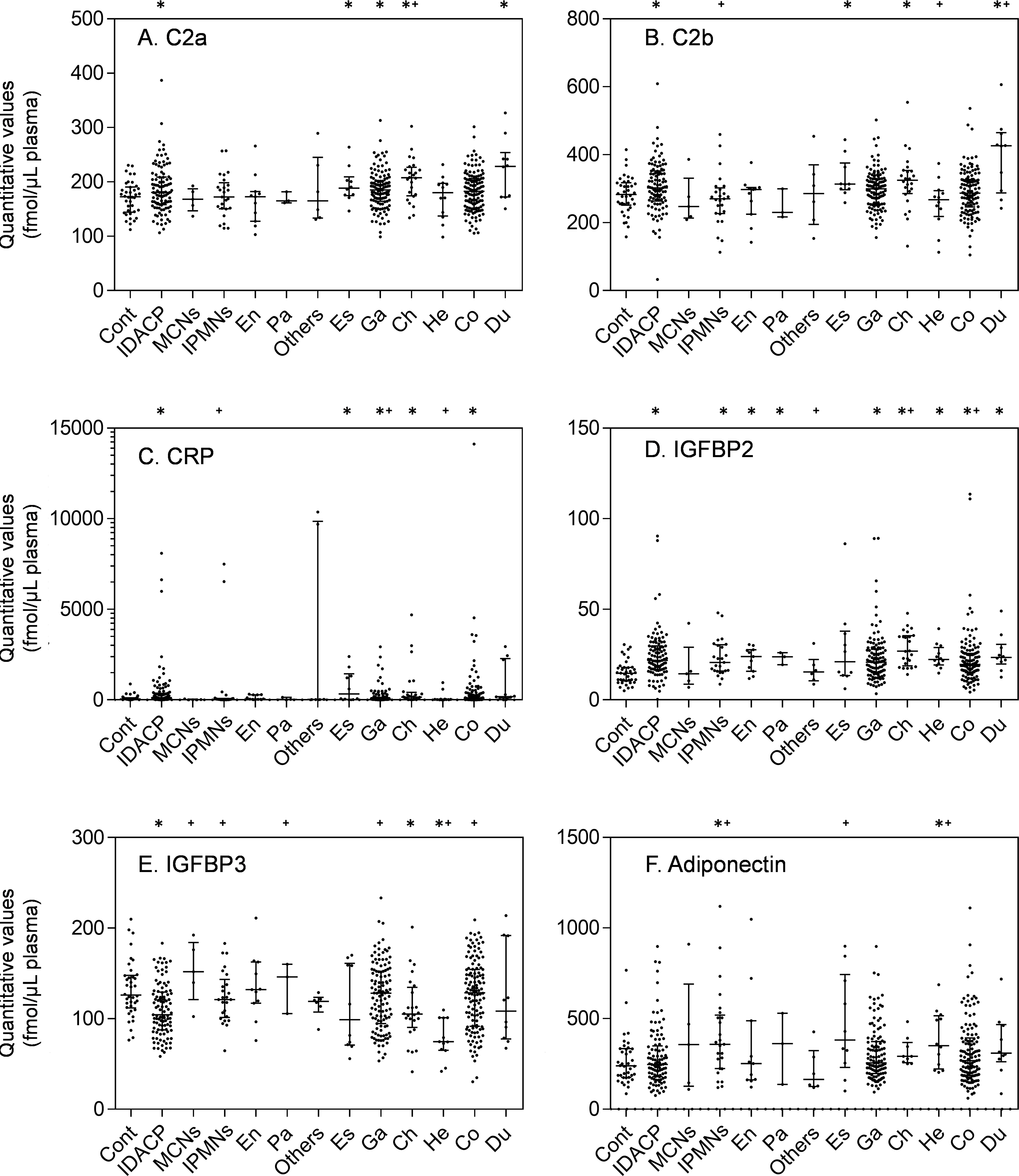

Supplement: S3 Fig — Lines represent median and quartiles. Healthy controls, cont; endocrine neoplasms, En; pancreatitis, Pa; esophageal cancer, Es; gastric cancer, Ga; cholangiocarcinoma, Ch; hepatocellular carcinoma, He; colon cancer, Co; duodenal cancer, Du. *, p<0.05 compared to healthy controls; ⁺, p<0.05 compared to IDACP. (TIF) [file pone.0161009.s003.tif]

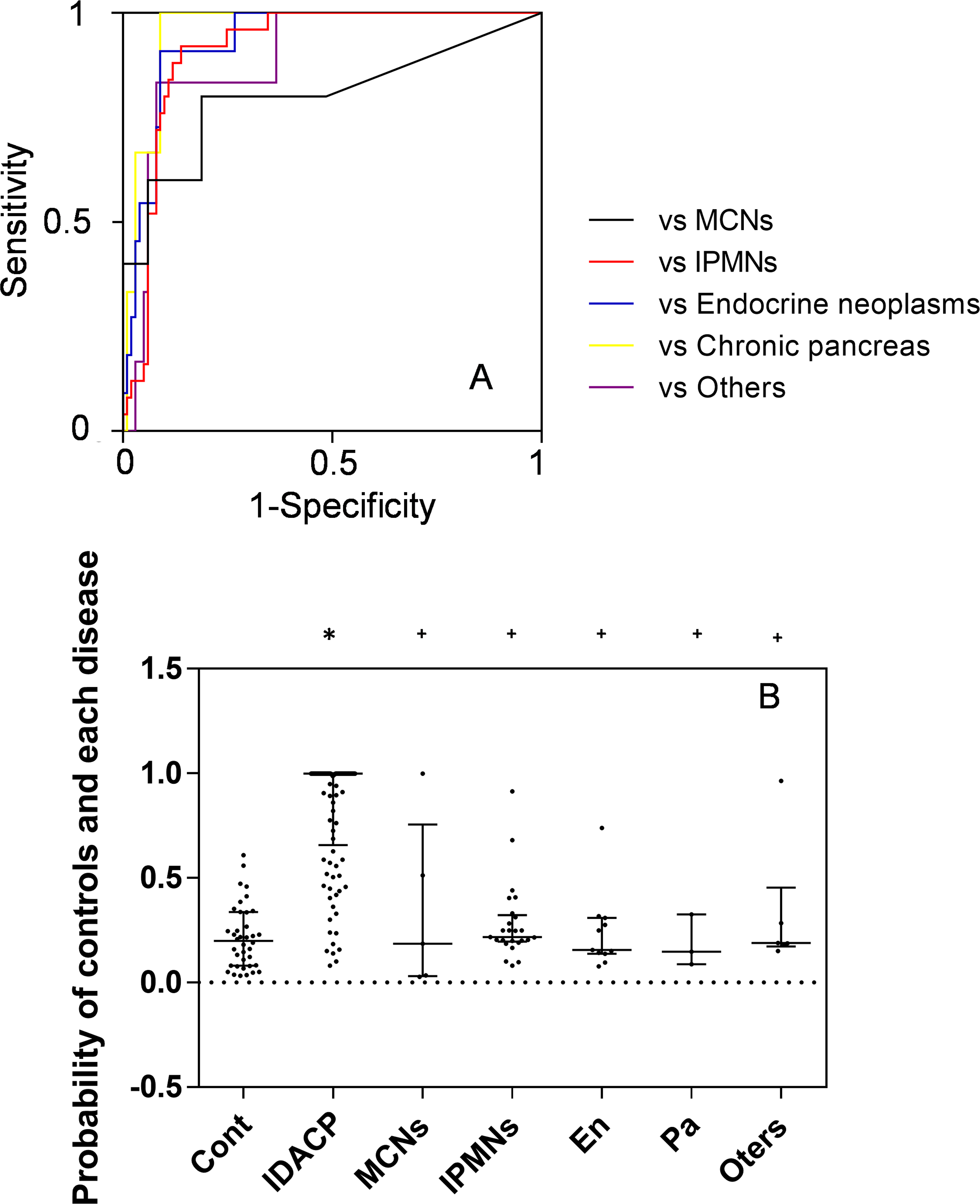

Supplement: S4 Fig — (A) ROC curves of Eq 1 among IDACP, pancreatic diseases and healthy controls. AUC values and 95%CI values were shown in Table 6. (B) Dot plot of probability of IDACP, pancreatic diseases and healthy controls calculated from Eq 1. Lines represent median and quartiles.*, p<0.05 compared to healthy controls; ⁺, p<0.05 compared to IDACP. (TIF) [file pone.0161009.s004.tif]

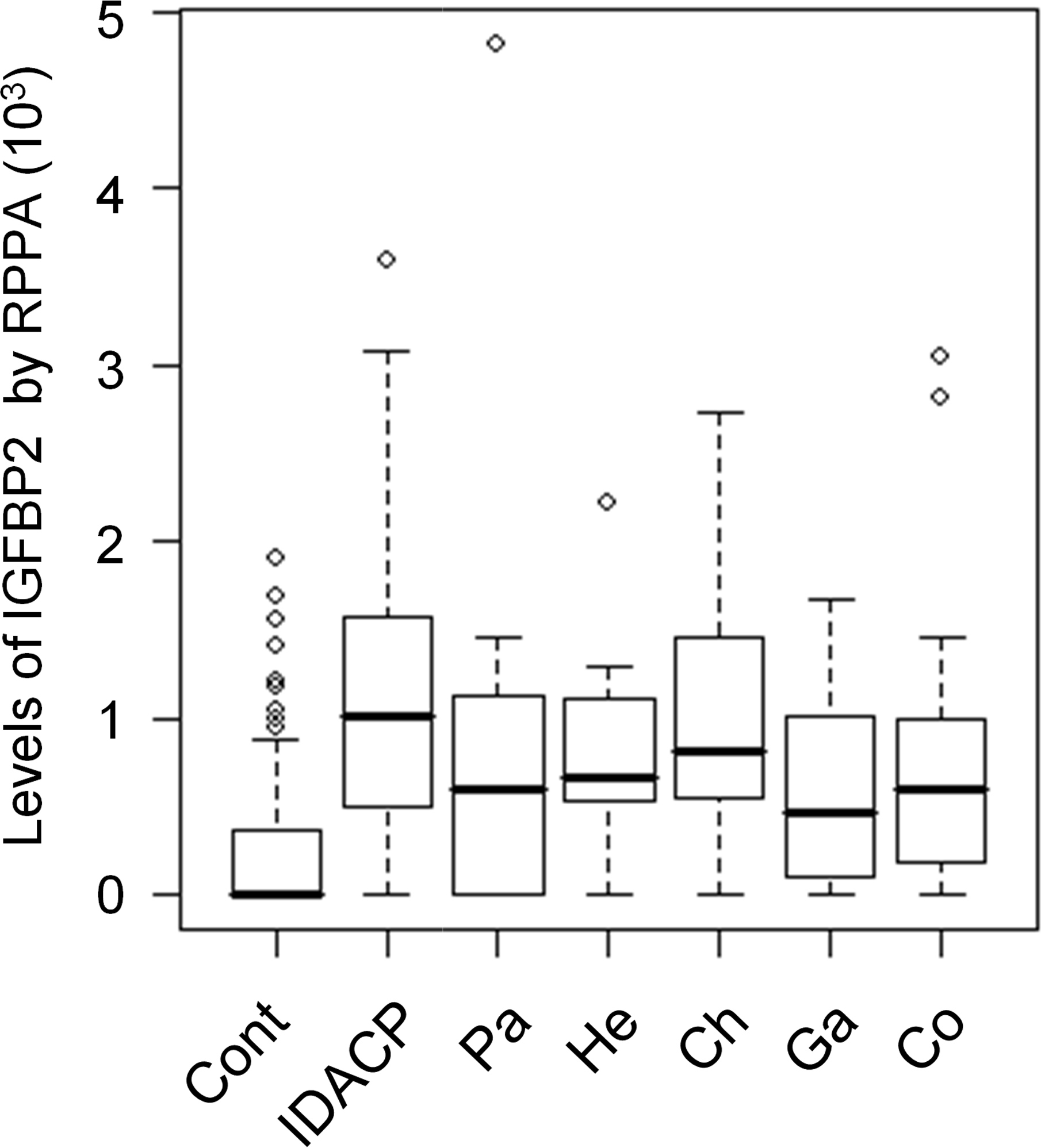

Supplement: S5 Fig — Healthy controls, cont; pancreatitis, Pa; hepatocellular carcinoma, He; cholangiocarcinoma, Ch; gastric cancer, Ga; colon cancer, Co. (TIF) [file pone.0161009.s005.tif]
