# Supplementary material for: Identification of IGFBP2 and IGFBP3 As Compensatory Biomarkers for CA19-9 in Early-Stage Pancreatic Cancer Using a Combination of Antibody-Based and LC-MS/MS-Based Proteomics
Source: PLoS One. 2016 Aug 31;11(8):e0161009. doi: 10.1371/journal.pone.0161009 (PMC5007017; doi:10.1371/journal.pone.0161009)
Supplement: S1 Table — The peptides for each target protein for LC-MS/MS analysis were selected by using in silico criteria [25,26]. The conditions of SRM/MRM were optimized for high signal intensity following direct injection of peptide solution into the mass spectrometer through a turbo ion spray source. Theoretical m/z values of doubly or thirdly charged ions of intact peptides (Q1) were assumed as precursor ions. Four singly or doubly charged fragment ions produced from precursor ions were selected as Q3-1, -2, -3 and -4. Bold letters with asterisks show the stable isotope-labeled amino acid residues (13C and 15N). (PDF) [file pone.0161009.s006.pdf]

S1 Table. SRM/MRM transitions

Protein name	Peptide sequence	SRM/MRM transition (*m/z*)			
		Q1/Q3-1	Q1/Q3-2	Q1/Q3-3	Q1/Q3-4
23 biomarker candidates for IDACP selected by RPPAs					
AK3L1	TLGQAEALDK	523.3 / 831.4	523.3 / 646.3	523.3 / 446.3	523.3 / 375.2
	TLGQAEALD**K***	527.3 / 839.4	527.3 / 654.4	527.3 / 454.3	527.3 / 383.2
ANXA6	ALIEILATR	500.3 / 702.4	500.3 / 573.4	500.3 / 815.5	500.3 / 460.3
	ALIEILAT**R***	505.3 / 712.4	505.3 / 583.4	505.3 / 825.5	505.3 / 470.3
AP3B1	LLDSITVPVAR	592.4 / 442.3	592.4 / 957.5	592.4 / 643.4	592.4 / 541.3
	LLDSITVPVA**R***	597.4 / 452.3	597.4 / 967.5	597.4 / 643.4	597.4 / 551.4
ATP6S1	NVLLFLQDK	545.3 / 876.5	545.3 / 763.4	545.3 / 327.2	545.3 / 650.4
	NVLLFLQD**K***	549.3 / 884.5	549.3 / 771.4	549.3 / 327.2	549.3 / 658.4
C2 (C2b)	SSGQWQTPGATR	638.3 / 501.3	638.3 / 916.5	638.3 / 730.4	638.3 / 602.3
	SSGQWQTPGAT**R***	643.3 / 511.3	643.3 / 926.5	643.3 / 740.4	643.3 / 612.3
CD82	EDSLQDAWDYVQAQVK	947.9 / 573.3	947.9 / 1136.6	632.3 / 573.3	632.3 / 672.4
	EDSLQDAWDYVQAQV**K***	952.0 / 581.3	952.0 / 1144.6	635.0 / 581.3	635.0 / 680.4
CKS1B	YDDEEFEYR	633.3 / 987.4	633.3 / 614.3	633.3 / 338.2	633.3 / 743.3
	YDDEEFEY**R***	638.3 / 997.4	638.3 / 624.3	638.3 / 348.2	638.3 / 753.3
CKS2	YFDEHYEYR	441.2 / 579.7	441.2 / 506.2	441.2 / 630.3	441.2 / 338.2
	YFDEHYEY**R***	444.5 / 584.8	444.5 / 511.2	444.5 / 640.3	444.5 / 348.2
CSPG2	YTLNFEAAQK	592.8 / 807.4	592.8 / 920.5	592.8 / 693.4	592.8 / 417.2
	YTLNFEAAQ**K***	596.8 / 815.4	596.8 / 928.5	596.8 / 701.4	596.8 / 425.3
CYCS	YIPGTK	339.7 / 402.2	339.7 / 248.2	339.7 / 277.2	339.7 / 305.2
	YIPGT**K***	343.7 / 410.2	343.7 / 256.2	343.7 / 277.2	343.7 / 313.2
EVI1	HFTDSLK	424.2 / 285.1	424.2 / 347.2	424.2 / 563.3	424.2 / 257.1
	HFTDSL**K***	428.2 / 285.1	428.2 / 355.2	428.2 / 571.3	428.2 / 257.1
HMGB2	NYVPPK	359.2 / 341.2	359.2 / 244.2	359.2 / 440.3	359.2 / 278.1
	NYVPP**K***	363.2 / 349.2	363.2 / 252.2	363.2 / 448.3	363.2 / 278.1
HYOU1	DEPGEQVELK	572.3 / 450.2	572.3 / 245.1	572.3 / 899.5	572.3 / 217.1
	DEPGEQVEL**K***	576.3 / 454.3	576.3 / 245.1	576.3 / 907.5	576.3 / 217.1
IGFBP2	LIQGAPTIR	484.8 / 742.4	484.8 / 614.4	484.8 / 355.2	484.8 / 389.3
	LIQGAPTI**R***	489.8 / 752.4	489.8 / 624.4	489.8 / 355.2	489.8 / 399.3
MMP9	SLGPALLLLQK	576.9 / 952.6	576.9 / 476.8	576.9 / 448.3	576.9 / 614.4
	SLGPALLLLQ**K***	580.9 / 960.6	580.9 / 480.8	580.9 / 452.3	580.9 / 622.4
MST4	NNPPTLVGDFTK	651.8 / 537.8	651.8 / 229.1	651.8 / 212.1	651.8 / 1074.6
	NNPPTLVGDFT**K***	655.8 / 541.8	655.8 / 229.1	655.8 / 212.1	655.8 / 1082.6
MYBL2	EDNSLLNQGFLQAK	788.9 / 1018.6	788.9 / 905.5	788.9 / 346.2	788.9 / 663.4
	EDNSLLNQGFLQA**K***	792.9 / 1026.6	792.9 / 913.5	792.9 / 354.2	792.9 / 671.4

S1 Table. Continued

Protein name	Peptide sequence	SRM/MRM transition (*m/z*)			
		Q1/Q3-1	Q1/Q3-2	Q1/Q3-3	Q1/Q3-4
23 biomarker candidates for IDACP selected by RPPAs					
PI3	VPFNGQDPVK	550.8 / 501.3	550.8 / 197.1	550.8 / 904.5	550.8 / 643.3
	VPFNGQDPV**K***	554.8 / 505.3	554.8 / 197.1	554.8 / 912.5	554.8 / 651.4
PPM1B	NVIEAVYSR	525.8 / 837.4	525.8 / 724.4	525.8 / 595.3	525.8 / 327.2
	NVIEAVYS**R***	530.8 / 847.5	530.8 / 734.4	530.8 / 605.3	530.8 / 327.2
RNASE1	YPNC(ACM)AYR	472.2 / 390.7	472.2 / 683.3	472.2 / 780.3	472.2 / 569.2
	YPNC(ACM)AY**R***	477.2 / 395.7	477.2 / 693.3	477.2 / 790.3	477.2 / 579.2
RNASET2	SWPFNLEEIK	631.8 / 989.5	631.8 / 495.3	631.8 / 745.4	631.8 / 892.5
	SWPFNLEEI**K***	635.8 / 997.5	635.8 / 499.3	635.8 / 753.4	635.8 / 900.5
STMN1	DPADETEAD	481.7 / 205.1	481.7 / 829.3	481.7 / 758.3	481.7 / 284.1
	DPADETE**A***D	483.7 / 209.1	483.7 / 833.3	483.7 / 758.3	483.7 / 284.1
VRK2	LPIPFPEGK	499.3 / 674.4	499.3 / 430.2	499.3 / 324.2	499.3 / 787.4
	LPIPFPEG**K***	503.3 / 682.4	503.3 / 438.2	503.3 / 324.2	503.3 / 795.4
Additional proteins of biomarker candidates for IDACP					
C2 (C2a)	HAIILLTDGK	540.8 / 209.1	540.8 / 204.1	540.8 / 322.2	540.8 / 319.2
	HAIILLTDG**K***	544.8 / 209.1	544.8 / 212.1	544.8 / 322.2	544.8 / 327.2
CRP	ESDTSYVSLK	564.8 / 347.2	564.8 / 696.4	564.8 / 609.4	564.8 / 446.3
	ESDTSYVSL**K***	568.8 / 355.2	568.8 / 704.4	568.8 / 617.4	568.8 / 454.3
IGFBP1	ALPGEQQPLHALTR	511.0 / 673.9	511.0 / 460.3	511.0 / 807.5	511.0 / 597.3
	ALPGEQQPLHALT**R***	514.3 / 678.9	514.3 / 470.3	514.3 / 817.5	514.3 / 607.4
IGFBP3	YGQPLPGYTTK	612.8 / 876.5	612.8 / 666.3	612.8 / 559.3	612.8 / 221.1
	YGQPLPGYTT**K***	616.8 / 884.5	616.8 / 674.4	616.8 / 559.3	616.8 / 221.1
Adiponectin	IFYNQQNHYDGSTGK	591.3 / 756.3	591.3 / 829.9	591.3 / 674.8	591.3 / 261.2
	IFYNQQNHYDGSTG**K***	593.9 / 760.3	593.9 / 833.9	593.9 / 678.8	593.9 / 261.2
