# Supplementary material for: Identification of IGFBP2 and IGFBP3 As Compensatory Biomarkers for CA19-9 in Early-Stage Pancreatic Cancer Using a Combination of Antibody-Based and LC-MS/MS-Based Proteomics
Source: PLoS One. 2016 Aug 31;11(8):e0161009. doi: 10.1371/journal.pone.0161009 (PMC5007017; doi:10.1371/journal.pone.0161009)
Supplement: S2 Table — All antibodies were obtained from Abnova. (PDF) [file pone.0161009.s007.pdf]

S2 Table. List of antibodies for RPPAs

No	Catalog No	Product name
1	H00008546-B01	Anti-AP3B1 pAb
2	H00000717-B02	Anti-C2 pAb
3	H00001163-B01	Anti-CKS1B pAb
4	H00001164-B01	Anti-CKS2 pAb
5	H00001462-B01	Anti-CSPG2 pAb
6	H00054205-B01	Anti-CYCS pAb
7	H00003732-B01	Anti-CD82 pAb
8	H00005266-B01	Anti-Pl3 pAb
9	H00006035-B01	Anti-RNASE1 pAb
10	H00008635-B01	Anti-RNASET2 pAb
11	H00007444-B01	Anti-VRK2 pAb
12	H00023054-M01	Anti-NCOA6(1954-2063) mAb
13	H00000648-M02	Anti-BMI1(PCGF4) mAb
14	H00001277-M01	Anti-COL1A1(1021-1108)mAb
15	H00009360-M02	Anti-PPIG(13-107) mAb
16	H00001610-M01	Anti-DAO(119-218) mAb
17	H00050807-M01	Anti-DDEF1(1030-1129) mAb
18	H00001728-M01	Anti-NQO1 mAb
19	H00002043-M02	Anti-EPHA4(887-986) mAb
20	H00002122-M01	Anti-EVI1(952-1051) mAb
21	H00002305-M02	Anti-FOXM1(702-801) mAb
22	H00002625-M01	Anti-GATA3(103-200) mAb
23	H00002896-M01	Anti-GRN(494-593) mAb
24	H00028951-M04	Anti-TRIB2(254-343) mAb
25	H00008349-M06	Anti-HIST2H2BE(36-126) mAb
26	H00003148-M03	Anti-HMGB2 mAb
27	H000008091-M01	Anti-HMGA2(1-92) mAb
28	H00003217-M03	Anti-HOXB7(55-120) mAb
29	H00008519-M01	Anti-IFITM1 mAb
30	H00003614-M01	Anti-IMPDH1(201-300) mAb
31	H00011004-M01	Anti-KIF2C(1-100) mAb
32	H00008543-M02	Anti-LMO4 mAb
33	H00051765-M03	Anti-MST4 mAb(RP6-213H19.1)
34	H00004318-M03	Anti-MMP9 mAb
35	H00004605-M02	Anti-MYBL2(601-700) mAb
36	H00010525-M01	Anti-HYOU1(901-999) mAb
37	H00005163-M01	Anti-PDK1(203-302) mAb
38	H00005328-M01	Anti-PLAU(78-177) mAb
39	H00005495-M01	Anti-PPM1B mAb
40	H00005725-M01	Anti-PTBP1(45-144) mAb

S2 Table. Continued

No	Catalog No	Product name
41	H00005937-M02	Anti-RBMS1 mAb
42	H00005957-M01	Anti-RCV1(101-200) mAb
43	PAB3988	Anti-AK3L1 pAb
44	PAB1687	Anti-BMI1 pAb
45	PAB3539	Anti-CDH3 pAb
46	PAB4695	Anti-COL1A1 pAb
47	PAB1701	Anti-UBE2S pAb
48	PAB3007	Anti-EPHA4 pAb
49	PAB3427	Anti-FYN pAb
50	PAB3419	Anti-BTK pAb
51	PAB4472	Anti-HDGF pAb
52	PAB3605	Anti-HPCAL1 pAb
53	PAB4830	Anti-CD82 pAb
54	PAB2109	Anti-MST4 pAb
55	PAB4306	Anti-MGAT2 pAb
56	PAB4773	Anti-MLLT4 pAb
57	PAB4798	Anti-MMP9 pAb
58	PAB4158	Anti-PPM1B pAb
59	PAB2874	Anti-CSNK1A1 pAb
60	PAB2735	Anti-PRPS1 pAb
61	PAB2456	Anti-HTRA1 pAb
62	PAB1868	Anti-PSCA pAb
63	PAB3609	Anti-RCVRN pAb
64	MAB3537	Anti-FN1 mAb, clone 568
65	PAB8027	Anti-CYCS pAb
66	PAB12285	Anti-IFITM1 pAb
67	PAB12294	Anti-ITGB2 pAb
68	PAB10313	Anti-AHSG pAb
69	PAB11271	Anti-BIRC5 pAb
70	PAB10210	Anti-FN1 pAb
71	H00000133-B01P	Anti-ADM Purified pAb
72	H00000197-B01P	Anti-AHSG Purified pAb
73	H00009447-B01P	Anti-AIM2 Purified pAb
74	H00000205-B02P	Anti-AK3L1 Purified pAb
75	H00054443-B01P	Anti-ANLN Purified pAb
76	H00000397-B01P	Anti-ARHGDIB Purified pAb
77	H00023650-B01P	Anti-TRIM29 Purified pAb
78	H00000537-D01P	Anti-ATP6AP1 Purified pAb
79	H00010755-B01P	Anti-GIPC1 Purified pAb
80	H00000716-B01P	Anti-C1S Purified pAb

S2 Table. Continued

No	Catalog No	Product name
81	H00000800-D01P	Anti-CALD1 Purified pAb
82	H00009308-B01P	Anti-CD83 Purified pAb
83	H00000991-D01P	Anti-CDC20 Purified pAb
84	H00001001-B01P	Anti-CDH3 Purified pAb
85	H00065010-B01P	Anti-SLC26A6 Purified pAb
86	H00001281-D01P	Anti-COL3A1 Purified pAb
87	H00001460-B01P	Anti-CSNK2B Purified pAb
88	H00027338-B01P	Anti-UBE2S Purified pAb
89	H00002335-B01P	Anti-FN1 Purified pAb
90	H00002534-D01P	Anti-FYN Purified pAb
91	H00002781-B01P	Anti-GNAZ Purified pAb
92	H00003068-B01P	Anti-HDGF Purified pAb
93	H00003162-B01P	Anti-HMOX1 Purified pAb
94	H00003191-B01P	Anti-HNRPL Purified pAb
95	H00003485-B01P	Anti-IGFBP2 Purified pAb
96	PAB13498	Anti-COL3A1 pAb
97	MAB3580	Anti-COL3A1 mAb(clone8D1-8C7)
98	PAB6303	Anti-UBE2I pAb
99	PAB13272	Anti-STMN1 pAb
100	PAB14863	Anti-ANXA6 pAb
101	PAB14345	Anti-COL1A2 pAb
102	PAB9978	Anti-Beta amyloid pAb
103	H00003500-B01P	Anti-IGHG1 pAb
104	H00003535-B01P	Anti-IGL pAb
105	H00007850-B01P	Anti-IL1R2 pAb
106	H00003925-D01P	Anti-STMN1 pAb
107	H00004257-D01P	Anti-MGST1 pAb
108	H00004686-B01P	Anti-NCBP1 pAb
109	H00011196-B01P	Anti-SEC23IP pAb
110	H00005033-B01P	Anti-P4HA1
111	H00005119-B02P	Anti-PCOLN3 pAb
112	H00008000-B01P	Anti-PSCA pAb
113	H00005691-B01P	Anti-PSMB3 pAb
114	H00005831-D01P	Anti-PYCR1 pAb
115	H00009052-B01P	Anti-GPRC5A pAb
116	H00008490-D01P	Anti-RGS5 pAb
117	H00006574-M01	Anti-SLC20A1 mAb
118	H00001811-M01	Anti-SLC26A3(501-600) mAb
119	H00006627-B01P	Anti-SNRPA1 pAb
120	H00006715-D01P	Anti-SRD5A1 pAb

S2 Table. Continued

No	Catalog No	Product name
121	H00006876-M01	Anti-TAGLN mAb
122	H00007009-B01P	Anti-TEGT mAb
123	H00007072-B01P	Anti-TIA1 pAb
124	H00010103-M05	Anti-TSPAN1(110-211) mAb
125	H00011065-M01	Anti-UBE2C(70-179) mAb
126	H00007468-B01P	Anti-WHSC pAb
127	H00000537-M01	Anti-ATP6AP1(51-150) mpAb
128	PAB14920	Anti-PLAU pAb
129	PAB0283	Anti-TRIM29 pAb
130	MAB0059	Anti-ARHGDIB mAb, clone97A1015
