# Supplementary material for: Identification of IGFBP2 and IGFBP3 As Compensatory Biomarkers for CA19-9 in Early-Stage Pancreatic Cancer Using a Combination of Antibody-Based and LC-MS/MS-Based Proteomics
Source: PLoS One. 2016 Aug 31;11(8):e0161009. doi: 10.1371/journal.pone.0161009 (PMC5007017; doi:10.1371/journal.pone.0161009)
Supplement: S3 Table — The %CV values were obtained based on the 4 SRM/MRM transitions. (PDF) [file pone.0161009.s008.pdf]

S3 Table. The average and median %CV of early-stage set and all-stage set.

	%CV			
	Early-stage set		All-stage set	
	Average	Median	Average	Median
C2a	11.9%	11.2%	9.12%	8.28%
C2b	12.7%	12.8%	13.3%	12.6%
CRP	18.9%	13.3%	14.4%	12.1%
IGFBP2	17.3%	15.3%	20.8%	18.8%
IGFBP3	11.2%	10.5%	14.8%	14.0%
Adiponectin	14.2%	12.7%	13.0%	12.1%
