# Supplementary material for: Identification of IGFBP2 and IGFBP3 As Compensatory Biomarkers for CA19-9 in Early-Stage Pancreatic Cancer Using a Combination of Antibody-Based and LC-MS/MS-Based Proteomics
Source: PLoS One. 2016 Aug 31;11(8):e0161009. doi: 10.1371/journal.pone.0161009 (PMC5007017; doi:10.1371/journal.pone.0161009)
Supplement: S4 Table — Levels of markers in plasma of CA19-9-negative IDACP patients (all-stage set) are shown. The values marked in gray are above and below the thresholds for IGFBP2 and IGFBP3 shown in Table 4, respectively. (PDF) [file pone.0161009.s009.pdf]

S3 Table. Levels of markers in plasma of CA19-9-negative IDACP patients (all stages)

Stage	CA19-9 (U/mL)	CEA (ng/mL)	DUPAN-2 (U/mL)	IGFBP2 (fmol/uL)	IGFBP3
I	8.8	1.1	24	26.02	107.79
II	11.8	2.4	24	13.90	152.52
II	11	1.4	24	27.78	92.56
II	20	1.7	24	7.48	161.74
II	25.9	1	40	15.07	130.11
II	23.3	2.9	24	34.55	100.35
II	35.6	2.4	24	31.90	58.35
III	8.3	34.8	1100	29.14	130.11
III	1.1	2	1601	10.27	112.47
III	13.2	1.1	24	16.17	128.87
III	0.9	8.2	1601	13.77	62.88
IV	0.9	3	620	30.88	73.69
IV	18.4	2.7	88	33.75	118.41
IV	32.4	3.6	28	23.53	110.29
IV	19.8	4	24	87.89	97.90
IV	24.7	8.2	1601	29.83	92.16
IV	12.1	12	1601	34.44	106.37
IV	0.9	1.3	1100	13.90	120.10
IV	29.7	23.7	1601	33.04	121.33
IV	0.9	168	1601	10.21	78.43
IV	18.9	3.6	1601	24.61	67.30
IV	31.5	1.8	1601	14.10	72.77
IV	2.7	7.2	1601	37.32	163.69
